# Supplementary material for: A multicenter, randomized, parallel-group confirmatory study protocol to evaluate the efficacy of Soft Protector CPC, a novel oral mucosal protectant, in preventing oral mucositis and alleviating pain in patients with breast cancer
Source: PLoS One. 2026 Jun 4;21(6):e0350803. doi: 10.1371/journal.pone.0350803 (PMC13235912; doi:10.1371/journal.pone.0350803)
Supplement: S3 File — (PDF) [file pone.0350803.s003.pdf]

Study Protocol

Title

A multicenter, randomized, parallel-group confirmatory study protocol to evaluate the efficacy of Soft Protector CPC, a novel oral mucosal protectant, in preventing chemotherapy-induced oral mucositis and alleviating pain in patients with breast cancer

Principal Investigator

Kazuhiro Omori, DDS, PhD
Department of Periodontics and Endodontics, Division of Dentistry, Okayama University Hospital
Address:
Phone:

Version: 2.0
Date: July 31, 2025

Revision History

Page	Before Revision (ver 1.0)	After Revision (ver 2.0)	Reason for Revision
General	Date of creation: January 14, 2025 Version: 1.0	Date of creation: July 31, 2025 Version: 2.0	Version update
Cover Page	[Principal (Representative) Investigator]	[Coordinating Investigator]	Revision based on the amendment of the Clinical Trials Act
P.1 Section 2.Study Organization	[Principal (Representative) Investigator]	[Coordinating Investigator / Principal Investigator]	Revision based on the amendment of the Clinical Trials Act
P.1 Section 2. Study Organization	[Monitoring Supervisor]Contact:	[Monitoring Supervisor]Contact:	Change of contact number
P.3 Section 4. Study Methods	(3) Planned duration of participation per subject: 29–84 daysScreening: 1–28 daysComparative treatment period: 14, 21, or 28 daysStandard: 28 days regardless of group assignment	(3) Planned duration of participation per subject: 29–112 daysScreening: 1–56 daysComparative treatment period: 14, 21, or 28 daysStandard: 21 days regardless of group assignment	Schedule revision to accommodate chemotherapy delays due to poor patient condition and to align with typical 3-week (21-day) chemotherapy cycles
P.4 Section 4. Study Methods	(4) Details of interventionThe first chemotherapy visit after allocation was defined as	(4) Details of interventionThe day of the pre-chemotherapy visit for device placement is defined as Day 1.	Clarification of allowable chemotherapy start timing

Page	Before Revision (ver 1.0)	After Revision (ver 2.0)	Reason for Revision
	Day 1 (start of the comparative treatment period).	Chemotherapy is to be initiated within 3 days (Day 0 to +3) after device application.	
P.5 Section 4. Study Methods	Device re-filling procedure during treatment period, including re-filling after dropout.	Device re-filling procedure revised: in case of chemotherapy postponement, the device should be temporarily removed and re-applied according to the rescheduled chemotherapy date.	Clarification of procedure for re-filling after chemotherapy postponement
P.5 Section 4. Study Methods	The principal investigator requests device delivery from Sun Medical Co., Ltd.	The coordinating investigator requests device delivery from Sun Medical Co., Ltd.	Revision based on the amendment of the Clinical Trials Act
P.7 Section 5. Observation and Examination Items	Oral findings: number of teeth, denture use, periodontal parameters (if available), untreated teeth	Oral findings: number of teeth, denture use, presence/absence of periodontal examination, untreated teeth	Revision of examination items
P.8 Section 5. Observation and Examination Items	Description of AEs, diseases, and device malfunctions recorded in the eCRF	Added: Cases of AEs occurring before device removal due to chemotherapy postponement are also to be evaluated.	Specification of procedures for postponed chemotherapy
P.9 Section 5. Observation Schedule	See list of changes (p.24)	See list of changes (p.25)	Review of study schedule to accommodate chemotherapy

Page	Before Revision (ver 1.0)	After Revision (ver 2.0)	Reason for Revision
			delay and 21-day regimens
P.10 Section 6. Endpoints	Remarks: Evaluation points limited to “contact area between tooth and mucosa” (target site). Multiple lesion sites evaluated individually.	Remarks revised: Simplified to specify evaluation of target area and clarify that NRS pain evaluation is site-specific.	Clarification of description
P.10–11 Section 6. Endpoints	(2) Secondary endpoints table	Added notes: “Pain disappearance in CTCAE v5.0 is defined as Grade 0 (none). Grade 0 is not defined for mucositis.”	Addition of explanatory notes
P.10–11 Section 6. Endpoints	5) Prevention/improvement of onset and pain (by regimen subgroup analysis)	5) Prevention **or** improvement of onset and pain (by regimen subgroup analysis)	Clarification of terminology
P.10–11 Section 6. Endpoints	9) Technical validity of the procedure10) Patient preference toward intervention	Added new item: 9) Use of Episil Oral Liquid (patient diary)10) Technical validity of procedure11) Patient preference toward intervention	Addition of new secondary item (use of Episil recorded in diary)
P.12 Section 8. Statistical Analysis	Analysis population defined as all randomized subjects who received at least one treatment (ITT).	Defined three analysis sets: FAS, mFAS, and SAF, with detailed criteria for inclusion/exclusion.	Revision due to procedural update for chemotherapy postponement and re-filling
P.12 Section 8. Statistical Analysis	(2) Main endpoint analysis: defined prevention/improvement for Grades 1–2	Revised: includes Grade 3 in definition of improvement; deleted redundant line.	Correction and clarification of definitions

Page	Before Revision (ver 1.0)	After Revision (ver 2.0)	Reason for Revision
P.12–13 Section 8. Statistical Analysis	Secondary endpoints ④–⑩ listed	Added: Subgroup analysis, patient diary-based measures (analgesic use, steroid use, Episil use), and patient preference.	Structural update and harmonization
P.13 Section 8. Statistical Analysis	No mention of multi-site pain analysis	Added clarification on site-based evaluation, worst-grade analysis, and new definitions for CTCAE v5.0 mucositis and pain (prevention/improvement criteria).	Addition of supplementary explanation
P.16 Section 14. Risk–Benefit Assessment	Notification items listed as ④–⑤ with outdated terminology	Updated items ④–⑤ to align with current Clinical Trials Act wording.	Revision based on legislative amendment
P.17 Section 15. Data Storage and Disposal	Information transferred to "Principal Investigator" at Okayama University Hospital	Changed to "Coordinating Investigator" at Okayama University Hospital	Revision based on Clinical Trials Act
P.17 Section 16. Source Document Access	Principal Investigator and site investigators provide access to records	Added Coordinating Investigator as responsible party	Revision based on Clinical Trials Act
P.17–18 Section 17. Periodic Reports	Principal Investigator responsible for annual report	Changed to Coordinating Investigator as reporting party	Revision based on Clinical Trials Act

Page	Before Revision (ver 1.0)	After Revision (ver 2.0)	Reason for Revision
P.18 Section 18. Funding and COI	Funding, COI management, and insurance under Principal Investigator	Revised to Coordinating Investigator	Revision based on Clinical Trials Act
P.18 Section 20. Handling of Inquiries and Complaints	Principal (Representative) Investigator responsible	Coordinating Investigator, Principal Investigator, and Subinvestigators responsible	Revision based on Clinical Trials Act
P.19 Section 25. Response to Diseases/Device Malfunctions	No mention of chemotherapy postponement	Added statement specifying evaluation of events occurring before device removal due to chemotherapy delay	Specification of procedures for postponed chemotherapy
P.21 Section 25. Response to Diseases/Device Malfunctions	Reporting chain: Principal → Representative Investigator → CRB	Reporting chain revised: Principal → Coordinating Investigator → CRB	Revision based on Clinical Trials Act
P.21 Section 25. Response to Diseases/Device Malfunctions (Severity Table)	Old terminology	Updated terminology and structure per revised Clinical Trials Act	Revision based on Clinical Trials Act
P.21 Section 25. Response to Device Malfunctions	Notification to Representative Investigator	Notification to Coordinating Investigator	Revision based on Clinical Trials Act

Page	Before Revision (ver 1.0)	After Revision (ver 2.0)	Reason for Revision
P.21 Section 26. Compensation	Compensation provided through insurance of Principal Investigator	Compensation provided through insurance of Coordinating Investigator	Revision based on Clinical Trials Act
P.21–22 Section 27. Reporting of Non-Compliance	Principal Investigator as reporting party	Coordinating Investigator as reporting party	Revision based on Clinical Trials Act
P.22 Section 28. Study Completion and Discontinuation	Principal Investigator responsible for reporting and disclosure	Coordinating Investigator responsible for reporting and disclosure	Revision based on Clinical Trials Act

Table of Contents

1. Title ... *12*

2. Study Organization ... *12*

3. Background, Objective, and Significance ... *13*

4. Study Methods ... *14*

(1) Study Type and Design ... 14

(2) Study Outline (Refer to the Study Flowchart) ... 14

(3) Planned Participation Period per Subject ... 15

(4) Investigational Medical Device ... 15

(5) Management of the Investigational Device ... 17

(6) Concomitant Medications and Therapies ... 18

1. Permitted Medications / Therapies ... 18

2. Prohibited Medications / Therapies ... 19

(7) Case Registration and Randomization ... 19

1. Informed Consent, Preliminary Registration, and Assignment of Subject Code ... 19

2. Screening Examination Prior to Full Registration ... 19

3. Full Registration and Randomization ... 19

4. Method of Randomization ... 19

(8) Post-Study Care ... 20

(9) Use of Specimens and Data from Other Institutions ... 20

5. Observations and Assessment Items ... *20*

(1) Participant Background ... 20

(2) Evaluation and Examination Items ... 21

(3) Interview on General and Local Conditions ... 22

(4) Confirmation of Adverse Events, Illnesses, and Device Malfunctions ... 22

Notes on Study Assessments and Procedures ... 24

6. Evaluation Items ... *25*

(1) Primary Endpoint 26
(2) Secondary Endpoints 27
(3) Evaluations Not Required for Control Group 28
7. Target Sample Size and Rationale *29*
8. Data Compilation and Statistical Analysis *29*
(1) Analysis Populations 29
(2) Statistical Methods 29
9. Study Period *32*
10. Criteria for Participant Selection *32*
(1) Inclusion Criteria 32
(2) Exclusion Criteria 32
11. Study Background and Scientific Rationale *33*
12. Procedures for Obtaining Informed Consent *33*
13. Handling of Personal Information *34*
14. Anticipated Burden, Risks, and Benefits *35*
(1) Expected Benefits 35
(2) Anticipated Risks and Disadvantages 35
(3) Management of Adverse Events 35
(4) Protocol Amendments 35
(5) Criteria for Discontinuation 36
15. Storage and Disposal of Study Records and Data (Including Retention Period) *36*
16. Access to Source Documents *37*
17. Periodic Reporting *38*
18. Funding Source, Conflict of Interest, and Financial Relationships *38*
19. Public Disclosure of Study Information *39*
20. Participant Inquiries and Complaints *39*

21. Procedures for Proxy Consent ... ***40***

22. Informed Assent Procedures ... ***40***

23. Emergency Life-Threatening Situations ... ***40***

24. Financial Burden and Compensation for Participants ... ***40***

25. Management of Adverse Events, Device Malfunctions, and Related Procedures ***41***

(1) Definition of Adverse Events and Device Malfunctions ... 41

(2) Definition of Serious Adverse Events (SAEs) ... 41

(3) Severity Grading ... 41

(4) Assessment of Frequency ... 42

(5) Causality Assessment ... 42

(6) Outcome Classification ... 42

(7) Expectedness and Anticipated Events ... 43

(8) Responsibilities and Reporting of Serious Adverse Events ... 43

(9) Reporting of Device Malfunctions ... 44

26. Compensation for Health Injury ... ***44***

27. Reporting and Management of Protocol Deviations and Noncompliance ... ***44***

28. Study Completion and Discontinuation ... ***45***

(1) Study Completion ... 45

(2) Study Discontinuation ... 46

29. Management of Significant or Incidental Findings ... ***46***

30. Outsourcing of Study-Related Tasks ... ***46***

31. Future Use of Study Data and Specimens ... ***47***

32. Monitoring and Audit ... ***47***

(1) Monitoring ... 47

(2) Audit ... 47

33. Intellectual Property and Ownership ***.. 47***
34. References ***... 47***

1. Title

A Multicenter, Randomized, Parallel-Group Confirmatory Study to Evaluate the Efficacy of a Novel Oral Mucosal Protectant (Soft Protector CPC) in Preventing Oral Mucositis and Alleviating Pain in Patients Undergoing Chemotherapy for Breast Cancer

2. Study Organization

This study will be conducted under the following organizational structure.

Principal Investigator / Coordinating Investigator

- **Affiliation:** Department of Periodontology, Okayama University Hospital
- **Title:** Dentist
- **Name:** Kazuhiro Omori
- **Address:**
- **Phone:**

Site Investigators

- **Institution:** National Hospital Organization Shikoku Cancer Center, Department of Oral and Maxillofacial Surgery
    - **Title:** Dentist
    - **Name:** Kohei Furukawa
    - **Address:**
    - **Phone:**
- **Institution:** Miyagi Cancer Center, Department of Dentistry
    - **Title:** Dentist
    - **Name:** Masatoshi Usubuchi
    - **Address:**
    - **Phone:**
- **Institution:** Sagara Hospital, Department of Oral and Maxillofacial Surgery
    - **Title:** Dentist
    - **Name:** Tomofumi Hamada
    - **Address:**
    - **Phone:**

Administrative Office and Data Center

- **Affiliation:** Department of Periodontology, Okayama University Hospital
- **Address:**
- **Phone:**
- **Responsible Person:**

Monitoring Supervisor

- **Affiliation:** Project Management Office, Advanced Medical Research Center, Okayama University Hospital
- **Title:**
- **Name:**
- **Phone:**

Randomization and Statistical Analysis Supervisor

- **Affiliation:** Division of Data Science, Advanced Medical Research Center, Okayama University Hospital
- **Title:** Senior Research Manager
- **Name:** Michihiro Yoshida
- **Phone:**

3. Background, Objective, and Significance

In Japan's *Fourth Basic Plan for Cancer Control*, the Ministry of Health, Labour and Welfare emphasizes the importance of promoting appropriate supportive care to manage side effects, complications, and sequelae of cancer treatment. One major side effect of cancer therapy (chemotherapy and radiotherapy) is oral mucositis, which develops in approximately 40% of patients receiving chemotherapy and in nearly all patients receiving radiotherapy for head and neck cancers.
Once oral mucositis develops, patients experience severe pain associated with mucosal injury, leading to difficulty in oral intake, malnutrition, and loss of strength, which can reduce motivation for cancer therapy and markedly impair quality of life (QOL). Therefore, the *Fourth Basic Plan* also specifies as an individual goal the promotion of oral management for cancer patients through collaboration between physicians, dentists, and dental hygienists, emphasizing the importance of maintaining QOL through adequate nutrition and prevention or mitigation of treatment-related complications.

The causes of oral mucositis include not only the direct effects of anticancer agents and radiotherapy but also mechanical irritation from sharp tooth edges due to wear or caries progression and microbial infection resulting from reduced salivary secretion and biofilm accumulation.

Currently, management relies primarily on oral care performed by healthcare professionals (dental hygienists, nurses, etc.) and on mucosal protectants such as Episil® oral liquid, which patients apply directly to the affected area. However, challenges remain due to the shortage of human resources, the limited duration of Episil's protective effect, the need for frequent self-application, and difficulty reaching certain oral sites. Furthermore, these existing treatments do not address the physical irritation from teeth that exacerbates mucosal pain.

To address these limitations, a novel oral mucosal protectant device (Soft Protector CPC) has been developed and obtained medical device approval. This product aims to maintain QOL by allowing cancer patients to continue adequate oral intake and to complete chemotherapy according to schedule, thereby contributing to improved survival, cure rates, and quality of life, including work and daily activities.

This confirmatory multicenter randomized controlled trial (four participating institutions) is designed to evaluate the preventive and pain-relieving efficacy of Soft Protector CPC in patients undergoing chemotherapy for breast cancer. Participants will be randomly assigned to receive standard care (oral care ± Episil®) with or without the use of Soft Protector CPC. Oral mucositis will be assessed according to CTCAE version 5.0 and version 3.0, which are widely used internationally.

4. Study Methods

(1) Study Type and Design

This is a multicenter, prospective, open-label, randomized, parallel-group comparative study.

(2) Study Outline (Refer to the Study Flowchart)

After obtaining written informed consent, eligibility will be assessed according to inclusion and exclusion criteria. Following screening examinations, eligible participants will be formally enrolled and assigned to either the intervention group (receiving standard care plus the investigational device) or the control group (receiving standard care only).

After the assigned interventions are implemented, intergroup comparisons will be conducted for the primary and secondary endpoints.

(3) Planned Participation Period per Subject

29 to 112 days

- **Screening Period:** 1–56 days
- **Comparative Period:** 14, 21, or 28 days
    - The standard duration will be 21 days regardless of allocation.
    - If chemotherapy is administered in treatment cycles, the period will correspond to one cycle.
    - Minor adjustments due to outpatient schedules are permissible.
- **Continuous Period:** 14, 21, or 28 days
    - After completing the comparative period, participants continuing chemotherapy will receive both standard care (oral care, Episil® oral liquid, etc.) and the investigational device, irrespective of group assignment.

(4) Investigational Medical Device

① Overview

**Item**	**Description**
**Generic name**	Dental polymer-based temporary sealing material / Oral mucosal protectant
**Product name**	*Soft Protector CPC*
**Manufacturer / Distributor**	Sun Medical Co., Ltd.
**Approval / Certification No.**	30500BZX00107000
**Intended use**	To cover defective or sharp tooth surfaces for short periods to protect oral mucosa and, through gradual release of cetylpyridinium chloride (CPC), to suppress biofilm formation and prevent infection. The device also reduces mechanical irritation to the oral mucosa caused by carious lesions or sharp edges due to attrition. CPC has been shown to inhibit *Streptococcus mutans* growth in a

Item	Description
	concentration-dependent manner, thereby reducing biofilm formation on the cured surface.
Mode of use	Clean the tooth surface. Remove one single-use blister pack just before use. Using gloved fingers (preferably nitrile gloves that are easy to shape and non-sticky), directly apply the paste to the causative tooth. Remove excess material, ask the participant to occlude, adjust the shape, and initiate polymerization by light irradiation. If dislodgment is expected, bonding treatment should be applied before filling. Remove with an excavator or explorer if necessary. The device can remain in place for up to 30 days.
Properties / Specifications	Light-curable paste composed of urethane dimethacrylate, polymer, silica, photoinitiator, and cetylpyridinium chloride.
Mechanism	Upon light irradiation, the photoinitiator and co-initiator activate, initiating polymerization of monomer components and hardening the paste.
Amount per unit	200 mg per blister (for one application). The amount used depends on the number of target teeth.
Storage conditions	Store unopened in a clean environment at 1–30°C, away from heat, humidity, and direct sunlight. Shelf life: 3 years from manufacture (indicated on packaging).

② Intervention Procedures

The comparative period (Day 1) begins on the day the investigational device is applied prior to chemotherapy. Chemotherapy should be initiated within three days after device placement (Day 0–3). The evaluation timepoints are scheduled for Day 14, 21, or 28 of each treatment period, depending on the chemotherapy regimen.

After baseline assessments and interviews on Day 1 (before chemotherapy), interventions are conducted as follows:

1) Comparative Period – Intervention Group

(Standard care [oral care, Episil® oral liquid, etc.] + investigational device)

- Record the start date of the comparative period.

- In addition to conventional oral care and protectants, the investigational device is applied to teeth responsible for mucosal trauma (e.g., crowding, sharp edges due to fracture or attrition).
- If multiple candidate teeth exist, all will be filled and individually assessed.
- For procedural consistency, filling will be performed by the site investigator or co-investigator (dentist).
- The light-curing and bonding process follows the method described above.
- The device remains in place throughout the 14–28-day period.
- If chemotherapy is postponed, the device will be removed and reapplied in coordination with the rescheduled chemotherapy.
- After final evaluation (Day 14, 21, or 28), the device will be removed.
- If dislodged earlier, the date and time will be recorded.
- If continued application is deemed inappropriate or the participant wishes to discontinue, the device will be removed and final assessments performed (see Section 14.5 Discontinuation Criteria)

2) Comparative Period – Control Group

(Standard care [oral care, Episil® oral liquid, etc.] only)

- Record the start date of the comparative period.
- Participants receive only standard care as per routine practice (see Section 6 for concomitant treatments).
- After the comparative period evaluation, during the continuous period (Day 1–14/21/28), the investigational device will be applied using the same procedure as the intervention group.
- Device removal, dislodgment recording, and discontinuation criteria are handled identically to the intervention group.

(5) Management of the Investigational Device

The procedures for managing the investigational device used in this study are as follows:

1. **Device Delivery**
    - The Coordinating Investigator will request Sun Medical Co., Ltd. to deliver the investigational device.
    - Before the first delivery, each Site Investigator will prepare a *Device Management Log* to record the receipt and usage of the device at their site.

- Upon delivery, the designated Device Manager will verify the quantity and condition of the received devices, record the necessary details in the log, and thereafter manage the inventory.

2. **Storage and Handling**
   - Each Site Investigator will store and handle the device in accordance with the storage conditions specified in Section 4(4) of this protocol.
3. **Device Distribution**
   - Based on usage requests, the Device Manager will distribute the required number of devices and document the relevant information in the management log.
4. **Handling of Used Devices**
   - Each Site Investigator will record the date of use, quantity used, quantity returned, and subject identification code for used devices, and ensure proper disposal.
5. **Post-Study Device Management**
   - After study completion, each Site Investigator will verify that the quantities of unused and used devices match the records in the management log.
   - Any discrepancies will be investigated, and the reason will be documented in the log.
   - Once reconciliation is complete, remaining devices will be disposed of appropriately, and all disposal records will be archived together with the management log.

(6) Concomitant Medications and Therapies

1. Permitted Medications / Therapies

- Standard oral care focusing on moisturization, mucosal protectants (e.g., *Episil*® oral liquid), and mouth rinses.
- Chemotherapy for the primary disease (including oral or intravenous steroids, continuous anti-inflammatory analgesics, and G-CSF administration during hematologic suppression).
- Treatments for comorbidities or intercurrent illnesses during the study period.
- As-needed use of anti-inflammatory analgesics is permitted for symptom relief.
  - The preferred agents are loxoprofen sodium 60 mg tablets or acetaminophen 200–300 mg tablets, standardized per institution.
  - The same analgesic should be used consistently during the study period.
  - The name, dosage, and number of tablets taken as needed will be recorded in the patient diary.

- Analgesics for fever or cancer-related pain may be co-administered.

2. Prohibited Medications / Therapies

- Oral steroid ointments (except as rescue medication; the number of uses must be recorded).
- Narcotics or opioid analgesics.
- Non-urgent dental treatments.
- Radiotherapy to the head and neck region.

If any concomitant medication or therapy is used, its content and timing must be recorded.

Rationale: The allowance of concomitant anti-inflammatory analgesics aims to minimize patient discomfort and avoid undue burden.

(7) Case Registration and Randomization

1. Informed Consent, Preliminary Registration, and Assignment of Subject Code

The Site Investigator (or sub-investigator) will conduct a preliminary eligibility check under routine clinical care.

After providing a full explanation of the study, written informed consent will be obtained. The participant will then be preliminarily registered in the EDC system, and a subject identification code will be assigned.

2. Screening Examination Prior to Full Registration

After consent, the investigator will conduct screening tests according to the study schedule (see p.7).

Based on the results, eligibility will be confirmed—participants who meet all inclusion criteria and none of the exclusion criteria will be deemed eligible and fully registered in the EDC system.

3. Full Registration and Randomization

Following full registration in the EDC system, randomization information will be generated automatically through the same system, assigning each participant to either the intervention or control group.

4. Method of Randomization

Before the start of the study, the Randomization Supervisor will prepare a reproducible randomization table using computer-generated random numbers.

A stratified block randomization method will be applied, stratified by study site planned to conduct the comparative period.

The allocation ratio between the intervention and control groups will be **1:1**, based on the planned total sample size.

As this is an open-label study, detailed algorithmic parameters of the randomization will not be described in this protocol to prevent predictability of assignment and potential selection bias. Instead, they are specified in a separate *Randomization Specification Document*, which will remain confidential until all participants have been assigned.

(8) Post-Study Care

After completion of this study, the Site Investigator (or sub-investigator) will provide medical care deemed most appropriate for each participant, incorporating any findings or outcomes obtained from the study.

(9) Use of Specimens and Data from Other Institutions

Because this confirmatory study is a multicenter collaborative trial, patient data from the following participating institutions will be utilized:

- Shikoku Cancer Center
- Miyagi Cancer Center
- Sagara Hospital

5. Observations and Assessment Items

(1) Participant Background

- Age at consent, medical history*, comorbidities**, primary disease classification (breast cancer), drug/food allergies, smoking history, alcohol consumption, and body weight.
- Presence and site of oral mucositis lesions (upper/lower labial vestibule, dorsal/right/left/ventral/tip of tongue, buccal mucosa).
- Planned date of starting the comparative period (Day 1) and details of concurrent chemotherapy regimen (agents, cycle presence, cycle duration).
- History of prior radiotherapy (excluding head and neck region) and timing.
- Presence of oral candidiasis.

* **Medical history:** diseases clinically significant between 1 year prior to registration and enrollment, and other resolved conditions (≥ 1 year before registration) judged relevant to participant background.

** **Comorbidities:** diseases with ongoing symptoms at the time of registration.

(2) Evaluation and Examination Items

Screening Period

- Confirmation of participant background.
- Interview and physical examination (for study purposes).
- Hematology (RBC, hemoglobin, hematocrit, WBC, neutrophils, platelets) – performed within routine clinical care.
- Blood biochemistry (total protein, albumin, AST, ALT, Na, K, Cl, BUN, creatinine, CRP) – within routine clinical care.
- Pain and QOL scores:
    - Oral mucositis grading by CTCAE v5.0 (MedDRA/J v25.1) and CTCAE v3.0,
    - PRO-CTCAE #3a and #3b,
    - EORTC QLQ-OH15 #35,
    - Numerical Rating Scale (NRS) for pain at affected sites.
- Presence, site, and onset of oral mucositis.
- Oral findings (number of remaining teeth, presence of dentures, periodontal examination performed or not, untreated teeth).
- Intraoral photographs.
- Panoramic radiograph.
- Confirmation of concomitant drugs/therapies.
- ECOG Performance Status (PS).
- G-CSF administration (yes/no).

Comparative Period

- Interview and physical examination.
- Hematology (as above).
- Blood biochemistry (as above).
- Pain and QOL scores (CTCAE v5.0 / v3.0, PRO-CTCAE #3a, 3b, EORTC QLQ-OH15 #35, pain NRS).
- Presence, site, and timing of oral mucositis.
- Presence and timing of oral candidiasis.
- Intraoral photographs.
- Confirmation of concomitant medications/therapies.

- Evaluation of procedural factors.
- Patient preference or acceptability of the investigational treatment.
- ECOG PS.
- Patient diary entries (once daily) including:
    - Device dislodgement (presence/absence),
    - Pain intensity (PRO-CTCAE #3a, #3b; EORTC QLQ-OH15 #35; pain NRS),
    - Number of as-needed anti-inflammatory analgesic tablets,
    - Number of uses of oral steroid ointment as rescue medication,
    - Use of *Episil*® oral liquid (yes/no).
- Adverse events.
- Device malfunctions.
- G-CSF administration (yes/no).

Continuous Period

Assessments identical to those conducted in the comparative period.

(3) Interview on General and Local Conditions

General and local physical conditions will be confirmed through interviews, and ECOG PS will be evaluated at each visit.

(4) Confirmation of Adverse Events, Illnesses, and Device Malfunctions

All adverse events, illnesses, and device malfunctions will be recorded in the electronic Case Report Form (eCRF), including details, onset/resolution dates, severity, treatment, outcome, seriousness assessment, and causal relationship to device use.

Follow-up investigations will be performed as needed.

Events occurring prior to device removal due to chemotherapy delay will also be included in safety evaluations.

Symptoms or disease progression attributable to the primary malignancy or to anticancer therapy itself will not be considered adverse events in this study.

For participants allocated to the control group during the comparative period, assessment of device-related malfunctions or causality is not applicable.

Schedule

Period	Screening period [a] / Enrollment	Comparative period			Continuous period: When chemotherapy is administered after the comparative period, existing therapy is combined with the filling of the investigational device irrespective of group assignment.
Assessment date	—	Day of study procedure (Day 1) [g]	End of comparative period (Day 14 / 21 / 28)	(At discontinuation)	End of continuous period (Day 14 / 21 / 28)
Allowable range	Day -56 to Day -1 (before procedure)	Assessment date (Day 0)	Assessment window: Day -7 to +7	Discontinuation date (Day 0 to +1)	Assessment window: Day -7 to +7
re-screening for eligibility	○				
Obtaining informed consent	—				
Pre-registration and eligibility screening	●				
Verification of subject background	●				
Final registration and randomization	●				
Interview / Physical examination / ECOG PS	●	●[c]	●	●	●
Administration of G-CSF (yes/no)	●	●	●	●	●
Blood tests (hematology and biochemistry)	●[i]		●	●	●

Pain/QOL assessments	●	●[c]	●	●	●
Occurrence, site, and timing of oral mucositis	●	●	●	●	●
Occurrence and timing of oral candidiasis		●	●	●	●
Oral findings	●				
Intraoral photographs	●	●	●	●	●
Panorama X-ray[b]	●				
Confirmation of concomitant medications and therapies	●	●	●	●	●
Placement of the investigational device in the oral cavity[d]		●	●		●
Procedure-related assessment[e]			●	●	●
Patient preference for the investigational treatment			●	●	●
PRO		←	—	—	→
Adverse events[f]		←	—	—	→
Device malfunction[h]		←	—	—	→

Notes on Study Assessments and Procedures

- ○ indicates items performed before obtaining informed consent.
- ● indicates items performed after obtaining informed consent.

[a] The screening examination shall be conducted within 56 days prior to Day 1 of the comparative period.

[b] Regarding radiographic imaging (panoramic X-ray), if results from routine clinical practice or health checkups performed within 12 months prior to obtaining consent are available, these may be used as the screening examination data.

[c)] Assessments marked accordingly must be performed prior to the investigational procedure.

[d)] If the investigational device dislodges before the end of any treatment period, the date and time of dislodgement must be recorded in the patient diary.

[e)] For participants assigned to the control group during the comparative period, the specified assessments and examinations for the comparative period are not required.

[f)] Symptoms or disease progression related to the underlying breast cancer, as well as events directly caused by chemotherapy, are not regarded as adverse events in this study. Events occurring before device removal due to chemotherapy delay will still be evaluated. For participants in the control group during the comparative period, assessment of causal relationship between adverse events and device filling is not applicable.

[g)] The day on which participants visit the site for device application prior to chemotherapy shall be defined as Day 1 (start of the comparative period). Chemotherapy should be administered within 0–3 days after device application, and the actual chemotherapy date must be recorded. If chemotherapy is postponed, the device should be temporarily removed, and the device application date reset in accordance with the rescheduled chemotherapy.

[h)] Evaluate whether natural dislodgement, brushing-related dislodgement, meal-related dislodgement, or self-removal by the participant occurred. If such events occur before temporary device removal due to chemotherapy delay, they must also be included in the evaluation.

[i)] If hematology or biochemistry tests performed for routine clinical purposes within 56 days prior to obtaining consent are available, they may be used as the screening examination data.

6. Evaluation Items

The timing of assessments during the continuous period will be identical to that in the comparative period.

Note on Evaluations:

For the evaluation items *"Oral mucositis (CTCAE v3.0 subcriteria)"* and *"Pain NRS at the affected site"*, the efficacy of the investigational device is principally limited to the *designated affected site*—defined in inclusion criterion (3) as "a site where contact occurs between teeth and oral mucosa" (corresponding to the filled site in the intervention group).

Therefore, this site must be identified in advance on Day 1 for all participants, including the control group (which does not receive device filling).

If multiple affected sites are present, each must be assessed individually.

(1) Primary Endpoint

Oral mucositis severity based on CTCAE v3.0 subcriteria during the comparative period

Grade	Description
Grade 1<br>Erythema of the mucosa	• Mucosal erythema and edematous changes without erosion or ulcer formation.
Grade 2<br>Patchy ulcerations or pseudomembranes	• Patchy and localized, with each lesion measuring less than 30 mm in diameter.<br>• "Localized" refers to an area that does not extend across multiple sub-sites.<br>• Even when ulcers are present in multiple sub-sites, they are considered localized if they occur sporadically in each area.
Grade 3<br>Confluent ulcerations or pseudomembranes; bleeding with minor trauma	• Lesions extending over a wide area measuring 30 mm or more in diameter.<br>• Lesions spreading across multiple sub-sites (e.g., from the hard palate to the soft palate, or from the left to the right side of the tongue).
Grade 4<br>Tissue necrosis; significant spontaneous bleeding; life-threatening consequences	• Ulcers or pseudomembranes spreading throughout the entire oral cavity.<br>• Persistent bleeding from the ulcerative surface.<br>• Extensive presence of coagulum-like crusts (excluding bleeding associated with detachment of superficial crusts caused by surface dryness).
Grade 5<br>Death	

(Source: Oral Supportive Care Study Group, Manual for the Evaluation of Oral Mucositis in Cancer Therapy, 1st Ed., OSC3, 2015.)

- This CTCAE v3.0–based evaluation serves as the primary endpoint for the comparative period.
- In the continuous period, the same outcome will be treated as a secondary endpoint.
- When Grade 1 symptoms disappear, the condition will be recorded as "none (Grade 0 for convenience)".

Rationale:

CTCAE v3.0 is a standardized, objective, and internationally accepted index for the evaluation of oral mucositis severity.

(2) Secondary Endpoints

1. The same CTCAE v3.0–based oral mucositis evaluation conducted during the continuous period.
2. Mucositis oral according to **CTCAE v5.0 / MedDRA/J v25.1**.

**Grade**	**Description**
1	Asymptomatic or mild symptoms; intervention not indicated
2	Moderate pain or ulcer that does not interfere with oral intake; modified diet indicated
3	Severe pain; interfering with oral intake
4	Life-threatening consequences; urgent intervention indicated
5	Death

Oral pain according to CTCAE v5.0/MedDRA/J v25.1

**Grade**	**Description**
1	Mild pain
2	Moderate pain; limiting instrumental ADL
3	Severe pain; limiting self care ADL
4	-
5	-

- In oral pain (CTCAE v5.0), disappearance of pain is defined as “none (Grade 0)”.
- In oral mucositis (CTCAE v5.0), Grade 0 is not defined.

3. **Quality of Life (QOL) Assessment [Patient-Reported Outcomes]**
    - PRO-CTCAE v1.0 (Items #3a and #3b)
    - EORTC QLQ-OH15 (Item #35)
    - Pain NRS at affected site

      The *Pain NRS* will be derived from the patient diary and represents the maximum pain level during each treatment period.

      The scale ranges from **0 to 10**:
    - 0: no pain
    - 1–3: mild pain
    - 4–6: moderate pain
    - 7–10: severe pain (10 = “unbearable pain, worst ever experienced”)

4. **Occurrence site and timing of oral mucositis**
   Evaluated across all intraoral regions (upper/lower labial vestibule, tongue dorsum/right/left/ventral/tip, buccal mucosa, palate, others), and specifically at the designated affected site.
5. **Subgroup analyses** by chemotherapy regimen for preventive and pain-relieving efficacy.
6. **Chemotherapy completion status** (treatment adherence).
7. **Number of as-needed anti-inflammatory analgesic tablets** (from patient diary).
8. **Number of rescue uses of oral steroid ointment** (from patient diary).
9. **Use of Episil® oral liquid** (presence/absence; patient diary).
10. **Technical performance assessment**
    Evaluation of natural, brushing-related, eating-related, and self-induced device dislodgement.
11. **Patient preference for continued use of the investigational treatment**
    Evaluation of participants' willingness to continue using the device.

Timing of Assessments:

- Items 1–5: Screening / registration, Day 1 of the comparative period, end (or discontinuation) of comparative period, and end of continuous period.
- Items 6–11: End (or discontinuation) of comparative period, and end of continuous period.

Rationale for Endpoint Selection:

- Items 1–3 (excluding localized NRS): internationally standardized measures for QOL in oral mucositis.
- *Localized NRS*: captures pain limited to the specific mucosal site.
- Item 4: assesses overall oral mucositis occurrence.
- Items 5–9: necessary to evaluate efficacy of the investigational device.
- Item 10: required for assessing technical feasibility of device application.
- Item 11: necessary for evaluating patient acceptability and continued use.

(3) Evaluations Not Required for Control Group

For participants assigned to the control group during the comparative period, the following assessments are not required (but are required during the continuous period, if conducted):

- Technical performance evaluation
- Patient preference for investigational treatment
- Device malfunction assessment
- Causality assessment of adverse events related to device use

7. Target Sample Size and Rationale

Target sample size: 154 participants

- Intervention group (standard care + investigational device): 77
- Control group (standard care only): 77

Rationale:

In the exploratory trial, the proportion of improvement in oral mucositis at Day 15 was 58.3% (7/12) in the intervention group and 0.0% (0/6) in the control group.

Considering the small control group size in the exploratory study, the uncertainty of the estimated effect, and the allowance of concomitant use of existing mucosal protectants (which may enhance control group outcomes), as well as inclusion of *prevention of onset* as an endpoint in this confirmatory study, the expected improvement rates were conservatively assumed as 50% in the intervention group and 25% in the control group.

With a two-sided significance level of 5% and 90% power using Pearson's chi-square test, 77 participants per group (total n=154) are required.

Based on previous accrual rates (≥10 participants per year per site), this total sample size is feasible within the study period across the participating centers.

8. Data Compilation and Statistical Analysis

(1) Analysis Populations

Three analysis populations will be defined:

1. **Full Analysis Set (FAS):**
   All participants who were randomized, received treatment, and had at least one efficacy assessment (primary or secondary).
2. **Modified Full Analysis Set (mFAS):**
   Subset of the FAS excluding participants with major protocol deviations affecting efficacy evaluation. Analyses using mFAS will be conducted as needed.
3. **Safety Analysis Set (SAF):**
   All participants who were registered and received at least one treatment.

(2) Statistical Methods

1) Analysis of Demographic and Baseline Characteristics

Major background variables and related characteristics will be summarized by group.
For quantitative variables, summary statistics (n, mean, standard deviation, standard error, minimum, maximum, and quartiles) will be calculated.
For categorical variables, frequencies and percentages will be tabulated.

2) Analysis of the Primary Endpoint

Primary Endpoint:

Oral mucositis severity based on CTCAE v3.0 subcriteria during the comparative period.

Statistical Analysis:

- Cross-tabulation of each CTCAE grade at Day 1 versus subsequent evaluation points by treatment group.
- Estimation of the frequency, percentage, and two-sided 95% confidence interval (CI) of subjects achieving *prevention of onset* or *pain improvement* at each timepoint.
- Comparison between groups using Pearson's chi-square test (two-sided, $\alpha = 0.05$).
- Estimation of the between-group difference with a two-sided 95% CI.

Definitions:

- *Prevention of onset:* Absence of mucositis at the target site before and after the evaluation period ("none → none").
- *Pain improvement:* CTCAE v3.0 oral mucositis grade at baseline "Grade 1/2/3" → lower grade or "none" at the target site.

3) Analysis of Secondary Endpoints

Secondary Endpoints:

1. Same as the primary endpoint during the continuous period.
2. Oral mucositis and oral pain grades (CTCAE v5.0 / MedDRA/J v25.1).
3. QOL: PRO-CTCAE v1.0 (#3a, #3b), EORTC QLQ-OH15 (#35), Pain NRS at the affected site.
4. Site and timing of oral mucositis onset.
5. Subgroup analysis by chemotherapy regimen for prevention/improvement outcomes.
6. Chemotherapy completion status.
7. Use of concomitant as-needed anti-inflammatory analgesics (from patient diary).
8. Frequency of oral steroid ointment as rescue medication (patient diary).

9. Use of Episil® oral liquid (patient diary).
10. Technical performance assessment.
11. Patient preference for continuation of the investigational device.

Statistical Methods:

- For items (1)–(2), analyses identical to those for the primary endpoint will be applied.
- For item (5), subgroup analyses by chemotherapy regimen will be performed similarly.
- For other items:
  - Categorical data: same as primary analysis (frequencies, percentages, chi-square).
  - Continuous data: summary statistics (as above), estimation of intergroup differences and 95% CIs, and use of two-sample Wilcoxon or two-sample t-tests depending on distribution.
  - Analyses will also be performed on *change from Day 1*.
  - *Pain NRS* will be analyzed both as a continuous variable and as categorical (0 = none, 1–3 = mild, 4–6 = moderate, 7–10 = severe).
  - For participants with multiple affected sites, the worst score per period will be used.

Definition of Prevention and Improvement (CTCAE v5.0):

- *Prevention:* Absence of oral mucositis throughout evaluation period ("none → none").
- *Improvement:* Baseline Grade 1/2/3 → lower grade or "none" after evaluation.
- Absence of mucositis is defined per Secondary Endpoint 4 (overall oral mucosal status).

For Oral Pain (CTCAE v5.0):

- *Prevention:* "Grade 0 → Grade 0".
- *Improvement:* Baseline Grade 1/2/3 → lower grade or "Grade 0".

4) Safety Evaluation

Safety Endpoints:

1. Adverse events and illnesses.
2. Device malfunctions.
3. Laboratory tests.

Methods:

- Adverse events, illnesses, and device malfunctions will be summarized by participant and by event.
- Adverse events and illnesses will be coded using MedDRA/J, and device malfunctions using the Japanese Medical Device Failure Terminology.

- Events will be tabulated by System Organ Class (SOC) and Preferred Term (PT), and summarized by causal relationship and severity when appropriate.
- For laboratory tests, summary statistics (as in Section 2.1) will be calculated by group.

5) Significance Level and Confidence Coefficient

- Significance level: 5% (two-sided)
- Confidence coefficient: 95% (two-sided)

9. Study Period

From the date of publication on the Japan Registry of Clinical Trials (jRCT) following approval by the Certified Review Board until December 31, 2027.
Registration deadline: February 28, 2027.
jRCT (Japan Registry of Clinical Trials):
https://jrct.mhlw.go.jp/

10. Criteria for Participant Selection

(1) Inclusion Criteria

Participants must be able to understand the study and provide written informed consent, and must meet all of the following criteria:

1. Patients with breast cancer scheduled to receive chemotherapy (including molecular-targeted agents or immune checkpoint inhibitors) at a participating institution.
2. No clinical signs of oral candidiasis.
3. Presence of tooth–mucosa contact.
4. Age ≥18 years at the time of consent.
5. ECOG Performance Status 0–1.
6. Oral feeding is functionally possible.

(2) Exclusion Criteria

Participants meeting any of the following criteria will be excluded:

1. Receiving, previously received, or scheduled to receive radiotherapy to the head and neck region.
2. Having fewer than 10 remaining teeth.
3. Currently wearing dentures.
4. Known allergy to cetylpyridinium chloride (CPC) or resin materials.
5. Pregnant or possibly pregnant.
6. Using or planning to use medical narcotics, opioids, or oral steroid ointments (dexamethasone).
7. Scheduled to undergo dental treatment during the study period.
8. Unable or unwilling to complete self-assessment questionnaires under physician guidance.
9. Any other condition judged by the investigator to make the participant unsuitable due to clinical, social, or conflict-of-interest–related reasons.

11. Study Background and Scientific Rationale

This is a multicenter, prospective, open-label, stratified, randomized, parallel-group confirmatory study designed to evaluate the preventive and pain-relieving efficacy of the investigational oral mucosal protectant.
The target sample size was determined to ensure both scientific validity and feasibility within the planned study duration.

12. Procedures for Obtaining Informed Consent

The principal or sub-investigator will provide potential participants with the IRB-approved consent form and written information sheet, and will explain the study in full both verbally and in writing before obtaining written voluntary consent.
If new safety or efficacy information arises, or if protocol amendments could influence the participant's willingness to continue, updated information will be promptly provided. Revised consent and information documents will be submitted for Certified Review Board (CRB) approval prior to re-consent.

The consent document must include the following information:

1. Title of the specified clinical study, institutional approval, and notification to the Minister of Health, Labour and Welfare (MHLW).

2. Name of the medical institution and name/title of the principal investigator.
3. Reason for participant selection.
4. Expected benefits and risks of study participation.
5. Statement that participation is voluntary.
6. Explanation regarding withdrawal of consent.
7. Assurance that refusal or withdrawal will not result in disadvantageous treatment.
8. Procedures for public disclosure of study information.
9. Participant's right to access or review study-related materials and how to do so.
10. Protection of personal information.
11. Methods for storage and disposal of specimens and data.
12. Any sponsorship or involvement of manufacturers or other entities.
13. Procedures for handling inquiries and complaints.
14. Information on study-related costs.
15. Availability and comparison of alternative treatments.
16. Compensation and medical care in the event of health injury.
17. Details of Certified Review Board review and oversight.
18. Any other necessary items concerning the conduct of the study.

13. Handling of Personal Information

All study personnel will comply with the Declaration of Helsinki and the Clinical Trials Act (Japan).
Each participant will be assigned a unique study identification code.
A linkage file between personal identifiers and study codes will be created and securely password-protected.
Names and direct identifiers will be removed from all source data used for analysis.
This linkage file will be securely stored throughout the study to prevent leakage.
Publications and reports will not include any personally identifiable information such as names or birthdates
Data obtained in this study will not be used for any purpose other than those specified in the protocol.

14. Anticipated Burden, Risks, and Benefits

(1) Expected Benefits

Participation may provide potential benefit by preventing or alleviating oral mucositis-related pain associated with chemotherapy through the use of the investigational oral mucosal protectant.
After study completion, participants will continue to receive the most appropriate medical care as determined by the investigator, including consideration of study findings.

(2) Anticipated Risks and Disadvantages

Participation may involve increased clinical visit time and additional effort for patient-reported evaluations.
There is a possibility of experiencing diseases or device-related events as described in Section 25(7) "Predictable Events," which may require medical treatment such as outpatient or inpatient care.
Unexpected adverse events, permanent impairment, or even death cannot be completely ruled out.
Use of the investigational device provided by the sponsor will incur no cost to the participant.
In the event of serious health injury (death, or grade 1–3 permanent disability) directly attributable to study participation, compensation will be provided as described in Section 26, "Compensation for Health Injury."

(3) Management of Adverse Events

If an adverse event occurs, the investigator will immediately provide appropriate medical care, document the event in the medical record and eCRF, and inform the participant of the management plan.
If the investigational treatment is discontinued due to an adverse event, the participant will be promptly notified and managed appropriately.

(4) Protocol Amendments

Safety-related information will be continuously reviewed, and the protocol and consent documents will be amended if necessary.
All amendments must be approved in advance by the Certified Review Board (CRB).
Amendments will be submitted to MHLW as follows:

- **Substantial amendment:**
    1. *Notification of change in implementation plan* (Form No. 2, Ministerial Ordinance)
    2. *Revised implementation plan*
- **Minor amendment:**
    1. *Notification of minor change in implementation plan* (Form No. 3)
    2. *Revised implementation plan*

For minor administrative changes (e.g., investigator contact details, institutional name updates), notification to the CRB will be made within 10 days, accompanied by submission of Form No. 3 to MHLW.

(5) Criteria for Discontinuation

When continuation is deemed impossible, the investigator shall discontinue the participant's involvement, explain the reason if appropriate, and ensure that post-discontinuation medical care is provided without disadvantage.

Reasons for discontinuation include:

1. Participant requests to withdraw. (The reason will be confirmed if possible, but not required.)
2. Worsening of primary disease, chemotherapy-related symptoms, or requirement for disallowed concomitant therapy.
3. Occurrence of adverse events making continuation difficult.
4. Noncompliance with the protocol.
5. Ineligibility determined upon further observation after registration.
6. Any other reason judged by the investigator to make continuation inappropriate.

In case of treatment discontinuation:

If continuation of the investigational treatment is deemed impossible for the above reasons, treatment will be discontinued with an appropriate explanation to the participant.

Participants who have received the investigational device will continue safety follow-up observations even after discontinuation.

15. Storage and Disposal of Study Records and Data (Including Retention Period)

The principal investigator shall prepare and maintain records regarding:

1. Identification of each study participant.

2. Clinical care and testing performed for participants.
3. Details related to study participation.
4. Any other records necessary for the conduct of this study.

In this study, each participating institution will provide data to Okayama University Hospital, where the coordinating investigator is based.All data exchanges will be conducted so as to exclude personally identifiable information.

Each institution will manage personal information in accordance with its internal policies.

All collected data will be securely stored in a locked facility (for Okayama University Hospital: the Department of Periodontology, Graduate School of Medicine, Dentistry and Pharmaceutical Sciences) until five years after study completion, and will then be destroyed with appropriate safeguards for privacy.

Personal identifiers such as names, addresses, and dates of birth will be removed before storage.

Essential documents — including the study protocol, implementation plan, informed consent forms, summary report, correspondence with the Certified Review Board (CRB), monitoring documentation, participant code lists, CRFs, and any materials ensuring data reliability (as well as descriptions of the investigational device) — will be securely stored for the same period (five years post-study completion) and subsequently disposed of appropriately.

Electronic data will be permanently deleted, and paper records will be shredded.

16. Access to Source Documents

Source documents in this study include:

1. Records of participant consent and data disclosure.
2. Medical records, laboratory data, imaging results, registration data, and other records serving as the basis for case report forms.

The coordinating investigator, site investigators, and institutions shall make all clinical research–related records and source documents available for direct inspection by monitors, the CRB, or regulatory authorities as required.

17. Periodic Reporting

The coordinating investigator will submit an annual report on the study's implementation status to both the site administrators and the Certified Review Board (CRB), within two months after each one-year period following the public registration date on the Japan Registry of Clinical Trials (jRCT). The report will use Unified Form No. 5 and Annex Form No. 3.

The annual report shall include:

1. Number of participants enrolled.
2. Occurrence and outcomes of study-related diseases or adverse events.
3. Noncompliance with regulations or protocol and the corresponding corrective actions.
4. Evaluation of study safety and scientific validity.
5. Details of involvement by any pharmaceutical or medical device manufacturers.

Within **one month** after receiving CRB review comments, the coordinating investigator will submit the following to the MHLW using Annex Form No. 3:

1. Name of the CRB listed in the implementation plan.
2. CRB judgment regarding continuation or suspension of the study.
3. Number of participants enrolled.

The coordinating investigator will share the contents of periodic reports with all site investigators. Each site investigator shall promptly report the shared information to the administrator of their institution.

18. Funding Source, Conflict of Interest, and Financial Relationships

This study is supported by the following funding sources:

- Operational grants of the Department of Periodontology, Okayama University Hospital.
- Clinical Research Promotion Fund of Okayama University Hospital.
- Japan Agency for Medical Research and Development (AMED) through the "Project for Practical Application of Innovative Cancer Medical Care."
- Provision of the investigational device by Sun Medical Co., Ltd.

The coordinating investigator will disclose any conflicts of interest (COI) in accordance with the *"Guidance on COI Management under the Clinical Trials Act"* and obtain CRB approval.

Any updates or changes in institutional or personal COI will be reviewed and reported in each periodic report to the CRB.

Participants will not incur any additional costs for the use of the investigational device; all expenses remain within the scope of standard clinical care.

19. Public Disclosure of Study Information

This study is registered in the Japan Registry of Clinical Trials (jRCT).
Results obtained from this study will be published in the jRCT registry, presented at academic conferences such as the Japanese Breast Cancer Society and the Japanese Society for Supportive Care in Cancer, and submitted as a manuscript to a peer-reviewed journal specializing in oncology supportive care.

20. Participant Inquiries and Complaints

The coordinating investigator and all site investigators will respond promptly and appropriately to any inquiries, consultations, or complaints from participants or their representatives.
Inquiry Contacts:

Okayama University Hospital
Department of Periodontics and Endodontics, Faculty of Dentistry,
Kazuhiro Omori, DDS, PhD; Shogo Takashiba, DDS, PhD
Address:
Tel:

Patient Relations Office (for complaints)
Comprehensive Patient Support Center, Okayama University Hospital
Tel:
Email:

Collaborating Institutions:

- **Shikoku Cancer Center (Department of Oral and Maxillofacial Surgery)**
  Kohei Furukawa, DDS
  Address:
  Tel:

- **Miyagi Cancer Center (Department of Dentistry)**
  Masatoshi Usubuchi, DDS, PhD
  Address:
  Tel:
- **Sagara Hospital (Department of Oral and Maxillofacial Surgery)**
  Tomofumi Hamada, DDS, PhD
  Address:
  Tel:

21. Procedures for Proxy Consent

Proxy consent will **not** be obtained in this study.

22. Informed Assent Procedures

Not applicable to this study.

23. Emergency Life-Threatening Situations

Not applicable to this study.

24. Financial Burden and Compensation for Participants

The investigational device (novel oral mucosal protectant) will be provided by Sun Medical Co., Ltd. Participation in this study may slightly extend consultation time but will not impose significant additional burden.

No honorarium or financial compensation will be provided for study participation.

25. Management of Adverse Events, Device Malfunctions, and Related Procedures

(1) Definition of Adverse Events and Device Malfunctions

In this specified clinical trial conducted under the Japanese Clinical Trials Act, “adverse events” refer to any illness, disability, death, infection, or device malfunction that is suspected to be causally related to the conduct of the study.

In this study, the principal and sub-investigators will document all adverse events observed from the start of the comparative period until the end of the continuous treatment period.

Events that occur before device removal due to chemotherapy delay will also be included as evaluation targets.

(2) Definition of Serious Adverse Events (SAEs)

Serious adverse events are defined as those meeting any of the following criteria:

1. Death or life-threatening condition.
2. Other serious medical events, including:
    - (i) Events requiring hospitalization or prolongation of hospitalization.
    - (ii) Disability.
    - (iii) Conditions leading to potential disability.
    - (iv) Events of similar seriousness to the above.
    - (v) Congenital anomalies or defects in offspring.

(3) Severity Grading

Severity of adverse events will be determined using the Common Terminology Criteria for Adverse Events (CTCAE) v5.0, Japanese JCOG version.

For events not listed in CTCAE, the following classification will apply:

Grade	Description
**Grade 1 (Mild)**	Asymptomatic or mild symptoms; clinical or laboratory findings only; no treatment required.
**Grade 2 (Moderate)**	Minimal, local, or noninvasive intervention indicated; limitation of instrumental ADL.

Grade	Description
**Grade 3 (Severe)**	Medically significant but not immediately life-threatening; hospitalization or prolongation required; limitation of self-care ADL.
**Grade 4 (Life-threatening)**	Urgent intervention indicated.
**Grade 5 (Fatal)**	Death related to the event.

(4) Assessment of Frequency

For recurrent or intermittent events that appear to resolve and recur, the investigator may consider them as a single event if judged clinically continuous from onset to final resolution.

(5) Causality Assessment

The investigator will evaluate the relationship of each adverse event or malfunction to both the investigational device (novel oral mucosal protectant) and study procedures, taking into account:

- Temporal relationship to device use
- Underlying disease course
- Concomitant medications or therapies
- Procedural deviations or accidents

Causality will be classified as:

1. **Related** (reasonable possibility, cannot be ruled out), or
2. **Not related** (no reasonable causal relationship).

(6) Outcome Classification

All adverse events will be followed until resolution or stabilization, or until further follow-up is deemed unnecessary by the investigator.

Outcomes will be categorized as follows:

1. Recovered
2. Improved
3. Not recovered (unchanged or worsened)
4. Recovered with sequelae

5. Death
6. Unknown

(7) Expectedness and Anticipated Events

Expectedness will be assessed based on the product's approved labeling information or prior clinical experience.

If the nature, severity, or frequency is inconsistent with known data, it will be considered unexpected.

Possible adverse events and anticipated frequencies include:

Incidence ≤1%

1. Abdominal pain, diarrhea
2. Taste disturbance

Incidence ≤0.1%

3. Allergic reaction to mucosal protectant components
4. Microbial substitution phenomena
5. Pulpal disorders

Possible Device Malfunctions

6. Discomfort during device wear
7. Detachment from filling site
8. Accidental swallowing due to intraoral detachment
9. Aspiration due to intraoral detachment

(8) Responsibilities and Reporting of Serious Adverse Events

1. The principal/sub-investigator must take all necessary actions (explanation to participant, treatment, etc.) immediately upon recognizing an SAE.
2. The principal investigator must promptly report the event to the institutional administrator and the coordinating investigator.
3. The coordinating investigator will report to the Certified Review Board (CRB) using Unified Report Form No. 9 and share relevant information with investigators at other sites.
   Site investigators must promptly notify their institutional administrators upon receiving such information.

4. The coordinating investigator must also report any event meeting MHLW requirements using Supplementary Form No. 2-2 (for medical devices) to the Minister of Health, Labour and Welfare, following the timelines below.

Reporting Timelines

Predictability	Event	Report to CRB	Report to MHLW
**Unexpected**	Death / Life-threatening	Within 7 days	Within 7 days
	Other serious events	Within 15 days	Within 15 days
	Non-serious events	At next periodic report	—
**Expected**	Death / Life-threatening	Within 15 days	—
	Serious events	Within 30 days	—
	Non-serious events	At next periodic report	—

(9) Reporting of Device Malfunctions

If a device malfunction occurs, the sub-investigator must report it to the principal investigator, who will notify the institutional administrator and coordinating investigator.
If the malfunction may cause serious harm, the coordinating investigator shall report it to the Certified Review Board within 30 days of awareness.

26. Compensation for Health Injury

The study is covered by clinical trial insurance to compensate participants for any health injury related to study participation.
In the unlikely event of a serious health injury (death or permanent disability grade 1–3) attributable to participation, compensation (including medical expense reimbursement and indemnity) will be provided from the insurance policy maintained by the coordinating investigator.

27. Reporting and Management of Protocol Deviations and Noncompliance

If a violation of laws, regulations, or the study protocol (“noncompliance”) is identified, the principal investigator must report it immediately to the institutional administrator and the coordinating

investigator.

Sub-investigators must report to the principal investigator upon becoming aware of such events.

If the coordinating investigator identifies major noncompliance, an opinion must be sought from the Certified Review Board without delay.

Major noncompliance refers to any deviation that may affect:

- Participant rights or safety, or
- Integrity or reliability of study results.

Examples include failure to adhere to inclusion/exclusion or discontinuation criteria, or prohibited concomitant therapy.

Deviations due to unavoidable medical necessity (e.g., emergency risk mitigation) are excluded.

The coordinating investigator will implement corrective and preventive actions, share information with other investigators, and ensure recurrence prevention.

All instances of noncompliance and subsequent corrective measures will be included in the periodic report to the CRB.

28. Study Completion and Discontinuation

(1) Study Completion

When the period for data collection related to the primary endpoint described in the protocol is completed, the coordinating investigator will, in principle, prepare a Primary Endpoint Report within one year.

When all data collection related to all endpoints is completed, a Final Report and its summary will be prepared within one year and submitted to the Certified Review Board (CRB).

If the preparation timing for both reports coincides, the Final Report will serve as the Primary Endpoint Report.

The coordinating investigator shall submit the summary of the Primary Endpoint Report or Final Report to the Minister of Health, Labour and Welfare (MHLW) and disclose it on the Japan Registry of Clinical Trials (jRCT) within one month after receiving CRB comments.

When submitting the Final Report summary to MHLW, the study protocol and participant information sheet will be included.

Upon completion of either report, the coordinating investigator will promptly provide copies to the heads of participating institutions and inform them when public disclosure has occurred.

All collaborating site investigators will be notified of the publication, and each will in turn report it to their institutional administrator.

(2) Study Discontinuation

The coordinating investigator will review the feasibility of study continuation in any of the following circumstances:

1. Emergence of significant information regarding the quality, safety, or efficacy of the investigational device.
2. Difficulty in participant recruitment such that the target sample size cannot reasonably be achieved.
3. Achievement of the study objective before reaching the planned sample size or duration.
4. Direction from the CRB requiring major protocol changes that cannot be implemented.
5. Recommendation or directive for discontinuation by the CRB.

If discontinuation is decided, notification will be made within 10 days using Unified Form No. 11 to the CRB and Form No. 4 to the MHLW.

Even after submitting the discontinuation notice, the coordinating investigator will continue to fulfill reporting obligations (e.g., safety reports, periodic reports) until the official study completion.

Any changes in study progress status will be submitted as amendments to the implementation plan as required.

29. Management of Significant or Incidental Findings

If clinically or genetically important findings regarding the participant's health (including incidental findings) are obtained during the study, such information may be disclosed to the participant when deemed appropriate by the investigator.

30. Outsourcing of Study-Related Tasks

The statistical analysis of this study will be outsourced to Kazuyoshi Sando (Yui Medical Stat Co., Ltd.), a statistical collaborator.

Only anonymized data will be provided for this purpose.

Oversight of the contractor will be conducted through regular progress meetings and documentation review.

31. Future Use of Study Data and Specimens

Data obtained from this study may be used for future research, but such use will require prior approval from the ethics committee.

32. Monitoring and Audit

(1) Monitoring

Monitoring will be performed in accordance with a separate Monitoring Plan that defines the procedures, responsibilities, and frequency of monitoring activities.

(2) Audit

No independent audit is planned for this study.

33. Intellectual Property and Ownership

The investigational device and treatment evaluation methods used in this study are based on publicly available information, and no novel or inventive outcomes are anticipated.
However, if intellectual property arises, it will be managed appropriately under the Okayama University Regulations on Employee Inventions and Intellectual Property Management.
Participants will be informed of this policy in writing, and their consent will be obtained before participation.

34. References

1. *The Fourth Basic Plan for Cancer Control*, Ministry of Health, Labour and Welfare, Japan.
2. *Manual for Oral Mucositis Evaluation in Oral Supportive Therapy for Cancer Treatment*, 1st ed., OSC3, 2015.

3. Japan Agency for Medical Research and Development (AMED). *Project for Practical Application of Innovative Cancer Medical Care: Supportive/Palliative Care Methodology for Clinical Trials – Policy on Supportive and Palliative Care (Mucositis Section), ver.1.1, 2021.*
4. Sio TT, Le-Rademacher JG, Leenstra JL, Loprinzi CL, Rine G, Curtis A, Singh AK, Martenson JA Jr, Novotny PJ, Tan AD, Qin R, Ko SJ, Reiter PL, Miller RC. *Effect of Doxepin Mouthwash or Diphenhydramine-Lidocaine-Antacid Mouthwash vs Placebo on Radiotherapy-Related Oral Mucositis Pain: The Alliance A221304 Randomized Clinical Trial.* JAMA. 2019;321(15):1481–1490. doi:10.1001/jama.2019.3504.
5. Soga Y, Sugiura Y, Takahashi K, Nishimoto H, Maeda Y, Tanimoto M, Takashiba S. *Progress of oral care and reduction of oral mucositis—A pilot study in a hematopoietic stem cell transplantation ward.* Support Care Cancer. 2010;19(2):303–307. doi:10.1007/s00520-010-1002-y.
6. Nakagawa M, Higuchi T, Teraoka Y, Soga Y. *Four cases of oral mucosal damage associated with hematopoietic stem cell transplantation managed by local hydrogel wound protectant (Episil® Oral Liquid).* Jpn J Hematopoietic Cell Transplant. 2019;8(1):36–42.
7. *European Oral Care in Cancer (EOCC) Group Oral Care Guidance*, 1st Japanese edition, Japanese Society of Supportive Care in Cancer, Mucositis Committee.
8. *MASCC/ISOO Evidence-Based Clinical Practice Guidelines for Mucositis Secondary to Cancer Therapy*, Japanese Edition, MASCC/ISOO Mucositis Study Group, 2014.
9. Wei J, Wu J, Wang H, Wang B, Zhao T, Meng L, Dong L, Jiang X. *A Bioadhesive Barrier-Forming Oral Liquid Gel Improved Oral Mucositis and Nutritional Status in Patients With Head and Neck Cancers Undergoing Radiotherapy: A Retrospective Single Center Study.* Front Oncol. 2021;11:617392. doi:10.3389/fonc.2021.617392.
10. Cheng Y, Qin SK, Chen YP, Dong LH, Sun XD, Yu SY, Wu SK. *Local analgesic effect of a bioadhesive barrier-forming oral liquid in cancer patients with oral mucositis caused by chemotherapy and/or radiotherapy: a randomized multicenter, single-use, positive-controlled, open-label study.* Onco Targets Ther. 2018;11:8555–8564. doi:10.2147/OTT.S185915.
11. Hadjieva T, Cavallin-Ståhl E, Linden M, Tiberg F. *Treatment of oral mucositis pain following radiation therapy for head-and-neck cancer using a bioadhesive barrier-forming lipid solution.* Support Care Cancer. 2014;22(6):1557–1562. doi:10.1007/s00520-014-2117-3.
