# Supplementary material for: A multicenter, randomized, parallel-group confirmatory study protocol to evaluate the efficacy of Soft Protector CPC, a novel oral mucosal protectant, in preventing oral mucositis and alleviating pain in patients with breast cancer
Source: PLoS One. 2026 Jun 4;21(6):e0350803. doi: 10.1371/journal.pone.0350803 (PMC13235912; doi:10.1371/journal.pone.0350803)
Supplement: Attachment — Submitted filename: PONE-D-25-56784 Review MR.pdf [file pone.0350803.s005.pdf]

PLOS ONE

A multicenter, randomized, parallel-group confirmatory study protocol to evaluate the efficacy of Soft Protector CPC, a novel oral mucosal protectant, in preventing oral mucositis and alleviating pain in patients with breast cancer

--Manuscript Draft--

**Manuscript Number:**	PONE-D-25-56784
**Article Type:**	Study Protocol
**Full Title:**	A multicenter, randomized, parallel-group confirmatory study protocol to evaluate the efficacy of Soft Protector CPC, a novel oral mucosal protectant, in preventing oral mucositis and alleviating pain in patients with breast cancer
**Short Title:**	Efficacy of Soft Protector CPC for treating chemotherapy-induced oral mucositis in patients with breast cancer
**Corresponding Author:**	Kazuhiro Omori, DDS, PhD<br>Okayama University - Shikata Campus: Okayama Daigaku - Shikata Campus<br>Okayama, --None-- JAPAN
**Keywords:**	breast cancer; chemotherapy; Oral mucositis; Randomized controlled confirmatory trial; Soft Protector CPC
**Abstract:**	Oral mucositis is a frequent and debilitating adverse event observed in patients undergoing chemotherapy or radiotherapy. Current management strategies are limited in duration, require frequent application, and fail to address the mechanical irritation from teeth. A novel device, Soft Protector CPC, was developed to overcome these limitations. This multicenter, randomized, two-arm, open-label, confirmatory trial aims to evaluate the efficacy and safety of Soft Protector CPC in patients with breast cancer undergoing chemotherapy. A total of 154 participants will be randomly assigned in a 1:1 ratio to receive either oral care with Soft Protector CPC or oral care alone. The primary endpoint will be oral mucositis as assessed according to the Common Terminology Criteria for Adverse Events (CTCAE) v3.0 during the comparative treatment period. The secondary endpoints will include CTCAE v3.0 during the continuous treatment period, oral mucositis, pain (CTCAE v5.0), quality of life (Patient Reported Outcomes-CTCAE version 1.0 [PRO-CTCAE v1.0], the 15-item oral health questionnaire of the European Organisation For Research And Treatment Of Cancer [EORTC QLQ-OH15], and the pain Numeric Rating Scale), onset and site of mucositis, completion of chemotherapy, use of rescue medications, technical feasibility, and patient preference. The safety endpoints will include adverse events, device malfunction, and laboratory tests. This trial is expected to establish the clinical utility of the Soft Protector CPC for the prevention and management of oral mucositis, with the potential to improve the patients' quality of life and adherence to cancer therapy. This study was approved by the Clinical Research Review Board and registered with the Japan Registry of Clinical Trials, jRCTs062250005, on April 18, 2025.
**Order of Authors:**	Kazuhiro Omori<br>Kohei Furukawa<br>Masatoshi Usubuchi<br>Tomofumi Hamada<br>Tadahiko Shien<br>Michihiro Yoshida<br>Yuki Nakatsuka<br>Katsuyuki Hotta<br>Soichiro Ibaragi<br>Shogo Takashiba
**Additional Information:**	

Question	Response
**Financial Disclosure**<br><br>Enter a financial disclosure statement that describes the sources of funding for the work included in this submission. Review the submission guidelines for detailed requirements. View published research articles from *PLOS ONE* for specific examples.<br><br>This statement is required for submission and **will appear in the published article** if the submission is accepted. Please make sure it is accurate.<br><br>**Funded studies**<br>Enter a statement with the following details:<br>• Initials of the authors who received each award<br>• Grant numbers awarded to each author<br>• The full name of each funder<br>• URL of each funder website<br>• Did the sponsors or funders play any role in the study design, data collection and analysis, decision to publish, or preparation of the manuscript?<br><br>Did you receive funding for this work?	Yes
Please add funding details.<br>as follow-up to "**Financial Disclosure**<br><br>Enter a financial disclosure statement that describes the sources of funding for the work included in this submission. Review the submission guidelines for detailed requirements. View published research articles from *PLOS ONE* for specific examples.<br><br>This statement is required for submission and **will appear in the published article** if the submission is accepted. Please make sure it is accurate.	This research is supported by the Japan Agency for Medical Development (AMED) under grant number JP25ck0106033.

**Funded studies**<br>Enter a statement with the following details:<br>• Initials of the authors who received each award<br>• Grant numbers awarded to each author<br>• The full name of each funder<br>• URL of each funder website<br>• Did the sponsors or funders play any role in the study design, data collection and analysis, decision to publish, or preparation of the manuscript?<br><br>Did you receive funding for this work?"	
Please select the country of your main research funder (please select carefully as in some cases this is used in fee calculation).<br>as follow-up to "**Financial Disclosure**<br><br>Enter a financial disclosure statement that describes the sources of funding for the work included in this submission. Review the submission guidelines for detailed requirements. View published research articles from *PLOS ONE* for specific examples.<br><br>This statement is required for submission and **will appear in the published article** if the submission is accepted. Please make sure it is accurate.<br><br>**Funded studies**<br>Enter a statement with the following details:<br>• Initials of the authors who received each award<br>• Grant numbers awarded to each author<br>• The full name of each funder<br>• URL of each funder website<br>• Did the sponsors or funders play any role in the study design, data collection and analysis, decision to publish, or preparation of the manuscript?<br><br>Did you receive funding for this work?"	JAPAN - JP
**Competing Interests**	The authors received financial support from the Japan Agency for Medical Research

Use the instructions below to enter a competing interest statement for this submission. On behalf of all authors, disclose any competing interests that could be perceived to bias this work—acknowledging all financial support and any other relevant financial or non-financial competing interests.<br><br>This statement is required for submission and **will appear in the published article** if the submission is accepted. Please make sure it is accurate and that any funding sources listed in your Funding Information later in the submission form are also declared in your Financial Disclosure statement.<br><br>View published research articles from *PLOS ONE* for specific examples.<br><br>**NO authors have competing interests**<br><br>Enter: *The authors have declared that no competing interests exist.*<br><br>**Authors with competing interests**<br><br>Enter competing interest details beginning with this statement:<br><br>*I have read the journal's policy and the authors of this manuscript have the following competing interests: [insert competing interests here]*<br><br>* typeset	and Development (grant number JP25ck0106033). This study supported by San Medical Co., Ltd., for providing the investigational material (Soft Protector CPC).
**Ethics Statement**<br><br>Enter an ethics statement for this submission. This statement is required if the study involved:<br><br>• Human participants<br>• Human specimens or tissue<br>• Vertebrate animals or cephalopods<br>• Vertebrate embryos or tissues	This trial was approved by Clinical Research Review Board of Okayama University (approval number: CRB24-024).

• Field research<br><br>Write "N/A" if the submission does not require an ethics statement.<br><br>General guidance is provided below. Consult the submission guidelines for detailed instructions. **Make sure that all information entered here is included in the Methods section of the manuscript.**	

**Format for specific study types**<br><br>**Human Subject Research (involving human participants and/or tissue)**<br>• Give the name of the institutional review board or ethics committee that approved the study<br>• Include the approval number and/or a statement indicating approval of this research<br>• Indicate the form of consent obtained (written/oral) or the reason that consent was not obtained (e.g. the data were analyzed anonymously)<br><br>**Animal Research (involving vertebrate animals, embryos or tissues)**<br>• Provide the name of the Institutional Animal Care and Use Committee (IACUC) or other relevant ethics board that reviewed the study protocol, and indicate whether they approved this research or granted a formal waiver of ethical approval<br>• Include an approval number if one was obtained<br>• If the study involved *non-human primates*, add *additional details* about animal welfare and steps taken to ameliorate suffering<br>• If anesthesia, euthanasia, or any kind of animal sacrifice is part of the study, include briefly which substances and/or methods were applied<br><br>**Field Research**<br><br>Include the following details if this study involves the collection of plant, animal, or other materials from a natural setting:<br>• Field permit number<br>• Name of the institution or relevant body that granted permission	
**Data Availability**<br><br>Authors are required to make the data underlying their research findings fully available, without restriction.<br><br>If the manuscript reports pilot or preliminary results, the data underlying those results must be made available at the time of publication. A Data Availability	N/A - No results are reported

Powered by Editorial Manager® and ProduXion Manager® from Aries Systems Corporation

Statement describing where the data can be found is required at submission. Your answers to this question constitute the Data Availability Statement and will be published in the article, if accepted. When legal or ethical requirements prohibit public data sharing, authors must indicate how researchers may obtain access to the data. See the PLOS Data Policy and FAQ for detailed information.<br><br>**Important:** Stating 'data available on request from the author' is not sufficient. If your data are only available upon request, select 'No' for the first question and explain your situation in the text box.<br><br>For protocols without pilot or preliminary data, authors are strongly encouraged to state how they plan to share research data from their study when it is completed or published.<br><br>If your study includes pilot data, will they be made fully available without restriction?	
**Describe where the data may be found in full sentences. If you are copying our sample text, replace any instances of XXX with the appropriate details.**<br><br>• If **no pilot data are reported** in the manuscript, please enter the following: *No datasets were generated or analysed during the current study. All relevant data from this study will be made available upon study completion.*<br>• If the manuscript reports pilot data **held in a public repository**, include URLs, accession numbers or DOIs. If this information will only be available after acceptance, indicate this by ticking the box below. For example: *All XXX files are available from the XXX database (accession number(s) XXX, XXX.).*<br>• If the manuscript reports pilot data contained **within the manuscript and/or**	No datasets were generated or analyzed during the current study. Deidentified research data will be made publicly available upon study completion and publication.

**Supporting Information files**, enter the following:<br>*All relevant data are within the manuscript and its Supporting Information files.*<br>• If neither of these applies but you are able to provide **details of access elsewhere**, with or without limitations, please do so. For example:<br><br>*Data cannot be shared publicly because of [XXX]. Data are available from the XXX Institutional Data Access / Ethics Committee (contact via XXX) for researchers who meet the criteria for access to confidential data.*<br><br>*The data underlying the results presented in the study are available from (include the name of the third party and contact information or URL).*<br>• This text is appropriate if the data are owned by a third party and authors do not have permission to share the data.<br><br>For study protocols with data management or sharing plans, authors are encouraged to briefly describe in their Data Availability Statement how data they generate will be made accessible when the study is completed. For example:<br>• Deidentified research data will be made publicly available when the study is completed and published.<br><br>* typeset	
Additional data availability information:	

Powered by Editorial Manager® and ProduXion Manager® from Aries Systems Corporation

Manuscript

Click here to access/download;Manuscript;20251028_Protocol_SoftProtecto

STUDY PROTOCOL

A multicenter, randomized, parallel-group confirmatory study protocol to evaluate the efficacy of Soft Protector CPC, a novel oral mucosal protectant, in preventing oral mucositis and alleviating pain in patients with breast cancer

Short title: Efficacy of Soft Protector CPC for treating chemotherapy-induced oral mucositis in patients with breast cancer

Kazuhiro Omori[1, *], Kohei Furukawa[2], Masatoshi Usubuchi[3], Tomofumi Hamada[4], Tadahiko Shien[5], Michihiro Yoshida[6], Yuki Nakatsuka[6], Katsuyuki Hotta[6], Soichiro Ibaragi[7], Shogo Takashiba[1]

1 Department of Pathophysiology-Periodontal Science, Faculty of Medicine, Dentistry and Pharmaceutical Sciences, Okayama University, Okayama, 700-8525, Japan, **2** Department of Dentistry and Oral Surgery, Shikoku Cancer Center, Ehime, 791-0280, Japan, **3** Department of Dentistry, Miyagi Cancer Center, Miyagi, 981-1239, Japan, **4** Department of Dentistry and Oral

Surgery, Sagara Hospital, Kagoshima, 892-0833, Japan, **5** Department of Breast and Endocrinological Surgery, Okayama University Hospital, Okayama, 700-8558, Japan, **6** Center for Innovative Clinical Medicine, Okayama University Hospital, Okayama, 700-8558, Japan, **7** Department of Oral and Maxillofacial Surgery, Faculty of Medicine, Dentistry and Pharmaceutical Sciences, Okayama University, Okayama, 700-8525, Japan

*Corresponding Author

Kazuhiro Omori, DDS, PhD

Associate Professor

Department of Pathophysiology-Periodontal Science,

Faculty of Medicine, Dentistry and Pharmaceutical Sciences,

Okayama University

2-5-1 Shikata-cho, Kita-ku, Okayama, 700-8525, Japan.

E-mail: kazu@okayama-u.ac.jp

Abstract

Oral mucositis is a frequent and debilitating adverse event observed in patients undergoing chemotherapy or radiotherapy. Current management strategies are limited in duration, require frequent application, and fail to address the mechanical irritation from teeth. A novel device, Soft Protector CPC, was developed to overcome these limitations. This multicenter, randomized, two-arm, open-label, confirmatory trial aims to evaluate the efficacy and safety of Soft Protector CPC in patients with breast cancer undergoing chemotherapy. A total of 154 participants will be randomly assigned in a 1:1 ratio to receive either oral care with Soft Protector CPC or oral care alone. The primary endpoint will be oral mucositis as assessed according to the Common Terminology Criteria for Adverse Events (CTCAE) v3.0 during the comparative treatment period. The secondary endpoints will include CTCAE v3.0 during the continuous treatment period, oral mucositis, pain (CTCAE v5.0), quality of life (Patient Reported Outcomes-CTCAE version 1.0 [PRO-CTCAE v1.0], the 15-item oral health questionnaire of the European Organisation For Research And Treatment Of Cancer [EORTC QLQ-OH15], and the pain Numeric Rating Scale), onset and site of mucositis, completion of chemotherapy, use of rescue medications, technical feasibility, and patient preference. The safety endpoints will include adverse events, device malfunction, and laboratory tests. This trial is expected to establish the

clinical utility of the Soft Protector CPC for the prevention and management of oral mucositis, with the potential to improve the patients' quality of life and adherence to cancer therapy. This study was approved by the Clinical Research Review Board and registered with the Japan Registry of Clinical Trials, jRCTs062250005, on April 18, 2025.

Keywords

Breast cancer; Chemotherapy; Oral mucositis; Randomized controlled confirmatory trial; Soft Protector CPC

Introduction

Oral mucositis is a common and debilitating side effect in cancer patients undergoing chemotherapy or radiotherapy. It involves inflammation and ulceration of the oral mucosa, causing severe pain and affecting the patient's ability to eat, drink, and maintain oral hygiene. This condition can significantly reduce quality of life and may lead to treatment interruptions or dose reductions, potentially compromising cancer therapy outcomes [1–3]. Current management strategies for oral mucositis have limited effectiveness and effect duration. They often require frequent application and fail to address the mechanical irritation from teeth, which can exacerbate symptoms [4–6]. To overcome these limitations, novel approaches such as protective oral devices are being investigated to provide more comprehensive and longer-lasting relief for patients with chemotherapy-induced oral mucositis.

To address these issues, we developed a novel mucosal protectant, Soft Protector CPC, which has been approved as a medical device (Sun Medical Co., Ltd., approval no. 30500BZX00107000). The newly developed Soft Protector CPC differs from conventional therapies in that it targets the teeth responsible for pain rather than the ulcerated mucosal surfaces affected by oral mucositis. Furthermore, Soft Protector CPC exerts an inhibitory effect on biofilm accumulation

at its surface through the sustained release of cetylpyridinium chloride (CPC), an antimicrobial agent. Therefore, protective materials are expected to suppress the progression of dental caries and gingivitis/periodontitis.

A previous exploratory, randomized, controlled, two-arm, open-label trial was conducted at Okayama University Hospital without restrictions on cancer type (protocol registration no. jRCTs062220084). While patients with various cancers were enrolled, the majority were cases of breast cancer, in whom the trial demonstrated particularly favorable outcomes, including a notable pain-relieving effect on chemotherapy-induced oral mucositis.

Based on the favorable outcomes of our previous exploratory trial, which demonstrated promising pain-relieving effects of Soft Protector CPC, we designed this confirmatory clinical study to validate its preventive and therapeutic efficacy for oral mucositis in patients undergoing chemotherapy for breast cancer. In this multicenter, randomized controlled trial across four institutions, patients will be assigned to receive either oral care alone (primarily focused on moisturizing) or oral care with Soft Protector CPC. The incidence and severity of oral mucositis will be assessed using the internationally recognized Common Terminology Criteria for Adverse Events (CTCAE) versions 3.0

and 5.0. This study hypothesizes that the use of the Soft Protector CPC will significantly reduce the incidence and severity of oral mucositis compared to oral care alone.

Materials and Methods

Objective, overall design, and framework.

This randomized, controlled, two-arm, open-label confirmatory trial aimed to evaluate and confirm the efficacy and safety of Soft Protector CPC compared to oral care alone in preventing chemotherapy-induced oral mucositis and alleviating pain in patients with breast cancer. This trial was approved by the Clinical Research Review Board of the Okayama University (approval number: CRB24-024).

Registration of the protocol

The Japan Registry of Clinical Trials (jRCT) registration number of this protocol is jRCTs062250005 (https://jrct.mhlw.go.jp/latest-detail/jRCTs062250005).

Endpoints

Assessment Schedule (Fig. 1)

Written informed consent will be obtained from all participants by principal investigator and co-investigators, and eligibility will be confirmed according to the inclusion and exclusion criteria. The participants will be registered after pre-registration examinations (screening tests) and final confirmation of eligibility. Enrolled participants will be randomly assigned to either the intervention group (oral care with the application of the Soft Protector CPC, Fig. 2) or the control group (oral care only). Based on the allocation results, interventions will be initiated and assessments will be conducted at the end of the comparative treatment period (1st course). After completion of the comparative period, all participants, regardless of group assignment, will proceed to the continuous period (2nd course) in which the Soft Protector CPC will be applied in addition to oral care. Assessments will also be performed at the end of the continuous period. Each period will be generally set to 21 days; however, if chemotherapy is administered in cycles, the duration of one cycle (either 14, 21, or 28 days) will be used (Fig. 3). Notably, the CTCAE and the European Organization for Research and Treatment of Cancer (EORTC) that will be used as endpoints are internationally recognized indicators that have also been adopted in clinical guidelines for the management of oral mucositis [7,8].

Study Visit	V0	V1	V2	V3
Enrollment & Allocation		Post-allocation		
Study Period			Comparative	Continuous
Timepoints: Day (d)	-56d pre V1 ~ -1d pre V1	0d	14, 21, 28d(A)	28, 42, 56d(A)
**Enrolment**				
Informed consent	X			
Eligibility assessment	X			
Randomization	X			
**Interventions**				
Intervention group		X	→	→
Control group			X(B)	→
**Assesments**				
CTCAE v3.0 sub-criteria	X	X	X	X
CTCAE v5.0 Oral mucositis & Pain	X	X	X	X
PRO-CTCAE v1.0 Items: #3a, 3b	X	X	X	X
EORTC-QLQ-OH15 Item:#35	X	X	X	X
NRS	X	X	X	X
Blood test	X		X	X
Oral exam	X	X	X	X
Oral photo	X	X	X	X
Panorama x-ray	X			
PRO		X	→	→
Patient preference			X	X
Adverse events		X	→	→
Devise malfunctions		X	→	→

Fig. 1 SPRIT schedule of enrolment, interventions, and assessments. (A) Evaluations will be conducted in accordance with the chemotherapy administration schedule. (B) Following completion of all assessments during the comparative period, the Soft Protector CPC will be placed in the oral cavity. NRS; pain numerical rating scale, PRO; patient-reported outcome.

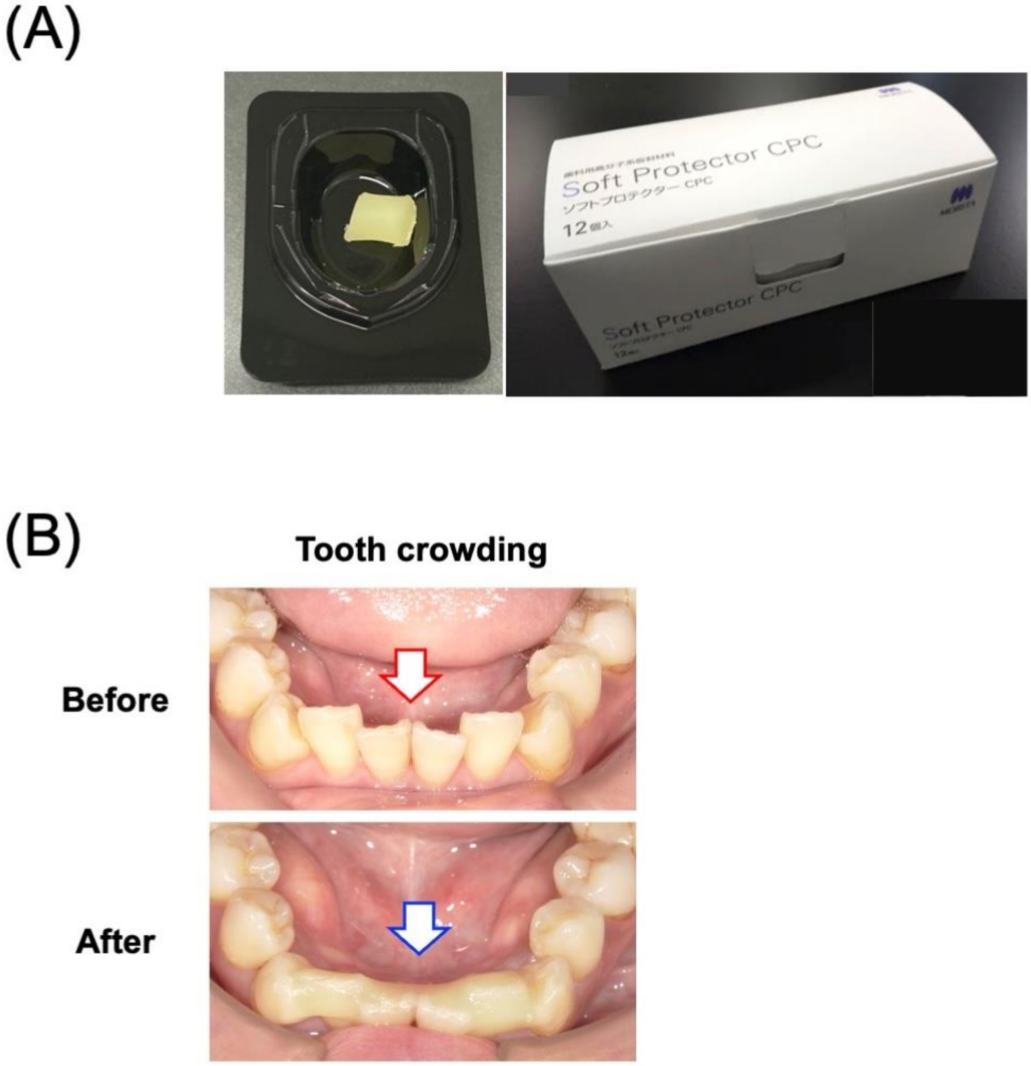

Fig 2. Soft Protector CPC. (A) A photograph of Soft Protector CPC, (B) A representative case for Soft Protector CPC.

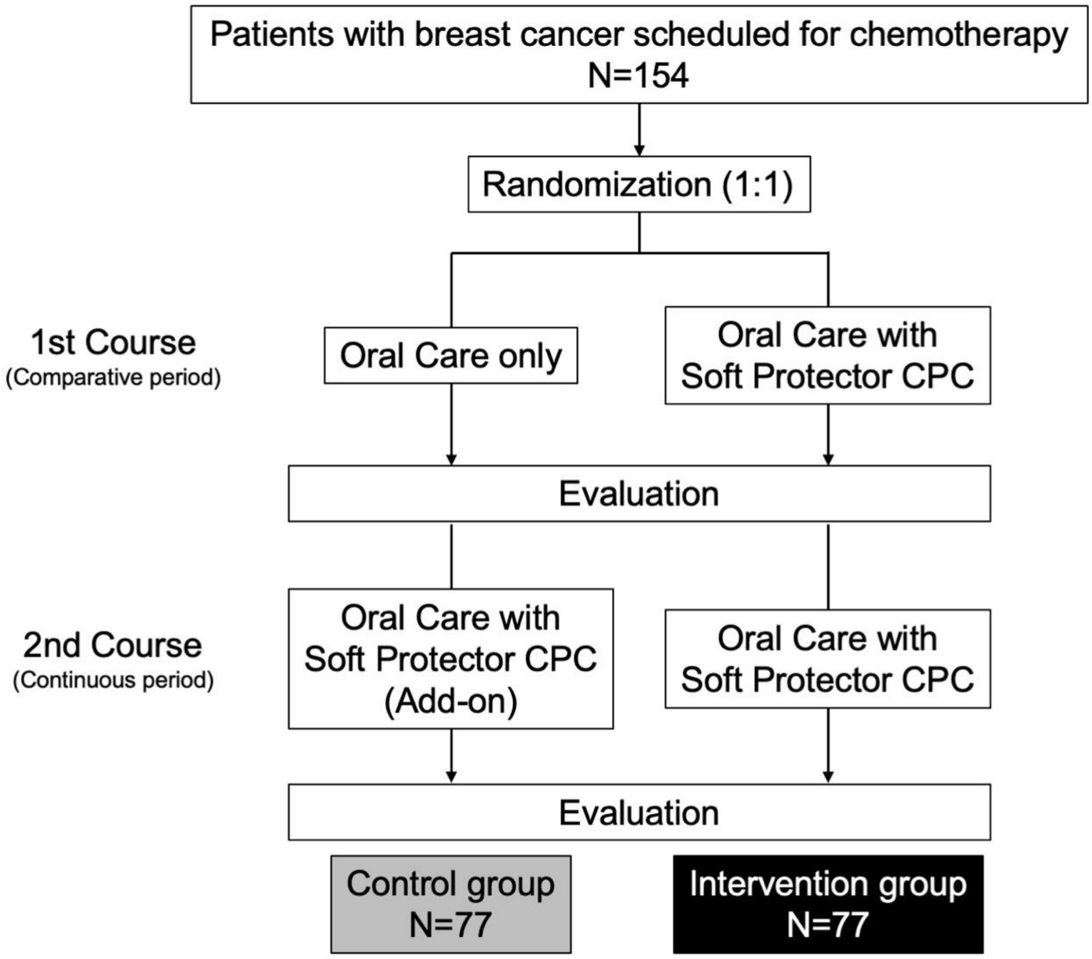

Fig 3. Validation study design.

Primary Endpoint

The primary endpoint in this study will be oral mucositis according to the subcriteria from the CTCAE v3.0 during the comparative period. Comparisons will be made before and after both the comparative and continuous periods; the primary endpoint will target the comparative period, whereas the

continuous period will be handled as a secondary endpoint. We will also evaluate the prevention of pain onset or the alleviation of pain. The endpoints are defined as follows:

- Prevention of onset: Absence of onset at the affected site both before and after the evaluation period.
- Alleviating pain: either a reduction in grade or an absence of onset at the affected site for cases of oral mucositis grade 1, 2, or 3 before the evaluation period.
- When the symptoms of grade 1 oral mucositis—according to the subcriteria from the Common Terminology Criteria for Adverse Events version 3 (CTCAE v3.0)–disappear, the patient’s condition is supplementarily defined as "absence of onset (for convenience, Grade 0)."

Secondary Endpoints

1. Oral mucositis according to the subcriteria from the CTCAE v3.0 during the continuous period. The assessment will be conducted in the same manner as for the primary endpoint. The evaluation will be performed during the continuous period.

2. Oral mucositis and oral pain grading (CTCAE v5.0 and the Japanese version of the Medical Dictionary for Regulatory Activities version 25.1 [MedDRA/J v25.1]). Comparisons will be performed for both the comparative and continuous periods, assessing the changes before and after each period. The same procedure will be applied to the subsequent endpoints. The terms and definitions relevant to oral mucositis and oral pain are as follows:
    - Prevention of onset: absence of the respective condition (mucositis or pain) in the entire oral cavity before and after the evaluation period.
    - Alleviating pain: reduction in grade or absence of the respective condition for cases with grade 1, 2, or 3 before the evaluation period.
    - When the grade 1 oral pain—as defined according to the CTCAE v5.0 and the MedDRA/J v25.1—disappears, it is supplementarily defined as "absence of onset (for convenience, Grade 0)." However, Grade 0 is not defined for the symptoms of oral mucositis.
3. Quality of life (patient-reported outcomes). PRO-CTCAE v1.0 (items: #3a, #3b), EORTC QLQ-OH15 (item: #35), and pain numerical rating scale (NRS) at the affected site will be used. The NRS of the maximum pain during each treatment period will be derived from

patient diaries. The NRS is scored from 0 to 10 (0 = no pain, 1–3 = mild pain, 4–6 = moderate pain, 7–10 = severe pain, with 10 being "the most intense pain ever experienced".

4. Occurrence and timing of oral mucositis: the presence of mucositis will be assessed across the entire oral cavity at each site (lips, tongue, buccal mucosa, palate, and other areas). Occurrence limited to the predefined affected sites will also be evaluated.
5. Subgroup analyses of pain alleviation or improvement of pain by the chemotherapy regimen.
6. Chemotherapy completion status.
7. Number of nonsteroidal anti-inflammatory drugs (NSAIDs), acetaminophen [paracetamol] or loxoprofen sodium) taken (patient-reported outcome [PRO]).
8. Frequency of oral steroid ointment use as rescue therapy (PRO).
9. Use of Episil® oral liquid (PRO) .
10. Technical feasibility (procedure-related assessments): spontaneous detachment, detachment during brushing or eating, and patient-initiated removal will be recorded.
11. Patient preference: patients will be asked whether they wish to continue the treatment with Soft Protector CPC.

Safety Endpoints

1. Adverse events
2. Device malfunctions
3. Laboratory tests

Eligibility Criteria

Inclusion Criteria

Participants must meet all the following criteria:

1. Patients with breast cancer undergoing chemotherapy, including molecular targeted therapy or immune checkpoint inhibitors at participating institutions.
2. Patients without oral candidiasis.
3. Patients with contact between the teeth and oral mucosa.
4. Patients aged ≥18 years at the time of consent.
5. An ECOG (Eastern Cooperative Oncology Group) Performance Status of 0 or 1.
6. Functionally capable of oral food intake.

Exclusion Criteria

Participants who meet any of the following criteria will be excluded:

1. Patients scheduled to undergo head and neck radiotherapy and those with a history of head and neck radiotherapy.
2. Patients with fewer than 10 teeth.
3. Patients who use dentures.
4. Patients with known allergies to CPC or resin.
5. Pregnant or potentially pregnant patients.
6. Patients using or scheduled to use medical narcotics, opioid formulations, or oral steroid ointments (e.g., dexamethasone).
7. Patients scheduled for dental treatment.
8. Patients who are unable or unwilling to complete the required assessments under physician supervision.
9. Patients deemed unsuitable for participation for clinical or social reasons or due to conflicts of interest

Randomization

Prior to study initiation, participants will be randomly assigned in a 1:1 ratio to the intervention and control groups. Randomization will be conducted using the stratified block method with study institutions as the stratification factors.

Because this is an open-label trial, describing the randomization algorithm in the protocol may increase the risk of predicting group assignment and introducing selection bias. Therefore, the detailed algorithm will be documented in a separate specification that will remain confidential until all the participants are assigned to a group.

Treatment

Intervention Group

Oral care plus Soft Protector CPC will be applied in both the comparative and continuous periods.

- In addition to oral care (mainly moisturizing), the investigational device will be applied to teeth that are likely to cause mucosal injury (e.g., crowding, sharp edges from fractures, or attrition). If multiple candidate teeth are present, all the teeth will be treated and individually evaluated.

- To ensure the consistency of the procedure, the application will be performed by the principal investigator or a co-investigator (dentist).
- The device, provided as a single-use blister packaging, will be removed from the package. After bonding (Hybrid-Coat II, Sun Medical Co. Ltd.), the device will be applied directly using gloved fingers. Excess material will be removed, occlusion will be confirmed, and polymerization will be initiated by light-curing until hardening.

Application and Management

- The device will remain in place during each treatment period (14, 21, or 28 days).
- If chemotherapy is postponed, the device will be removed and reapplied on the rescheduled date.
- At the end of each period, the device will be removed after the assessment.
- If the device is detached, the date and time will be recorded.
- If the device remains but continues to decline, adverse events arise, or the discontinuation criteria are met, the device will be removed for ethical and safety considerations, and end-of-treatment assessments will be performed.

Control group

During the comparative period, only oral care (primarily focused on moisturization) will be provided. After completion of the comparative period, the Soft Protector CPC will be applied during the continuous period, following the same procedure as in the intervention group. Removal and discontinuation will follow the same procedures as the intervention group.

Safety Monitoring

All severe adverse events will be recorded using the EDC. The principal investigator will compile the oral adverse events reported by the site investigators and submit them to the Clinical Research Review Board of Okayama University.

PRO

Patients will complete a paper-based PRO, including the weekly PRO-CTCAE v1.0 (#3a, #3b) and EORTC QLQ-OH15 (#35). In addition, patients will record daily whether the device

detached, pain NRS at affected sites, number of NSAIDs taken, and rescue use of oral steroid ointment and Episil® oral liquid.

Data Management

Data will be collected using the EDC system (UHCT ACReSS, Tokyo, Japan) by authorized personnel (principal investigator, sub-investigator, and research collaborators). Each participant will be assigned a study code, and de-identified data will be used for analysis. All hard copies were de-identified and stored securely at Okayama University. The data will be retained for five years following the completion of the study and then destroyed.

Sample size and statistical analyses

This study aims to confirm the superiority of oral care with Soft Protector CPC over oral care alone in preventing oral mucositis and alleviating the pain associated with this condition.

In a previous exploratory study, improvement of oral mucositis symptoms was evaluated according to the subcriteria from the CTCAE v3.0 after 15 days of Soft Protector CPC application. The results showed that 58.3% (7/12) achieved this endpoint with oral care plus Soft Protector CPC,

compared with 0.0% (0/6) with oral care alone. Considering the limited sample size in the exploratory study and the differences in study design between the exploratory study and this trial, the proportion of patients achieving prevention of oral mucositis or alleviation of pain in this study was assumed to be 50.0% in the intervention group and 25.0% in the control group. Using Pearson's chi-square test with a two-sided significance level of 5%, a sample of 154 patients (77 per group) was deemed necessary to achieve a statistical power of 90%. Considering the historical enrollment at the participating institutions, the planned sample size is feasible.

The primary endpoint will be the proportion of patients achieving prevention of the onset or alleviation of pain during the comparative period, and a 95% confidence interval will be calculated by treatment group. The superiority of Soft Protector CPC over the control treatment will be assessed using the Pearson's chi-square test with the statistical significance set at 5% (two-tailed).

Interim Analysis and Monitoring

No interim analyses will be performed. Monitoring will be conducted by designated monitors in accordance with institutional SOPs. On-site (site visits and source data verification) and off-site (telephone, fax, and email) monitoring will be performed.

- Before initiation: Ethics approval, trial registration, document storage, and device management.
- Informed consent: verification of approved forms, correct documentation, and secure data storage
- Patient registration: Screening, eligibility, and allocation procedures.
- During treatment: verification of oral mucositis assessments, required tests, device performance, and data consistency. Monitoring will be performed at least once every six months.
- Discontinuation/serious events: verification of the discontinuation criteria met and regulatory reporting.
- Completion: Confirmation of proper document archiving, device management, and termination procedures.

Dissemination

The results of this clinical trial will be presented at academic conferences, registered in a clinical trial registry (Japan Registry of Clinical Trials), and published in peer-reviewed journals.

Ethics approval and current status of this trial

The study protocol (ver. 1.0) was performed in accordance with the Declaration of Helsinki and the Pharmaceutical Affairs Act of Japan and was approved by the Clinical Research Review Boards of Okayama University Hospital, Shikoku Cancer Center, Miyagi Cancer Center, and Sagara Hospital. The start date was April 18, 2025, and the first patient completed the registration on May 29, 2025. Four patients have been enrolled until now and more patients are being recruited from four institutions as of September 10, 2025. We plan to enroll 154 participants in the full analysis set. The limit of enrollment will be February 28, 2027, and the planned study will end on December 31, 2027. In addition, the study protocol (ver. 2.0) was approved on October 28, 2025.

Discussion and Conclusion

This confirmatory trial, developed based on findings from a previous exploratory study, was designed to generate high-quality evidence confirming the efficacy of Soft Protector CPC, a newly developed oral mucosal protectant, in preventing and managing chemotherapy-induced oral mucositis in patients with breast cancer. In an exploratory trial, a high incidence of oral mucositis was observed

in patients undergoing chemotherapy for breast cancer. Previous reports have reported frequent occurrences of oral mucositis in patients receiving breast cancer chemotherapy regimens such as doxorubicin + cyclophosphamide (AC), docetaxel + cyclophosphamide (DC), and, more recently, datopotamab deruxtecan (Dato-DXd) [9–11]. The target population of the present study is patients with breast cancer. In addition, by focusing on both prevention and pain relief, and incorporating internationally recognized assessment tools (CTCAE, PRO-CTCAE, and EORTC QLQ-OH15) [7,8], this study addresses unmet clinical needs that are not fully covered by existing supportive care measures.

The strength of this study lies in its randomized multicenter design and emphasis on patient-reported outcomes, which are critical for evaluating both clinical efficacy and quality of life. Furthermore, this study comprises two periods: a comparison period and a continuous period. The The participants of both groups will have the opportunity to attach the Soft Protector CPC during the continuous period. The inclusion of technical feasibility and patient preference assessments is expected to provide valuable insights into real-world applicability.

However, some limitations of this study should be acknowledged. First, because the random assignment is whether the Soft Protector CPC is attached, the trial is designed in an open-label setting,

which may introduce bias in the evaluation. To minimize the influence of such bias, the primary endpoint was evaluated using the objective assessment criteria of oral mucositis, according to the subcriteria from the CTCAE v3.0. Furthermore, the study population was restricted to patients with breast cancer, and extrapolation to other cancer populations should be treated with caution. A small exploratory, randomized, controlled, two-arm, open-label study was conducted at Okayama University Hospital without restrictions of cancer type. As patient populations of different cancer types receive different chemotherapies and other treatments, we will prioritize improving the accuracy of statistical inference to confirm the effectiveness of this product using a reasonable sample size by specifying the cancer population.

Despite these limitations, the findings of this trial are expected to provide an evidence base for integrating the Soft Protector CPC into supportive care strategies, potentially improving quality of life and treatment adherence in patients with cancer.

Acknowledgements

We express our sincere gratitude to Dr. Masao Irie (Department of Biomaterials, Graduate School of Medicine, Dentistry and Pharmaceutical Sciences, Okayama University), Takashi Ito, Daisuke Uchida, and Jun Sakurai (Center for Innovative Clinical Medicine, Okayama University Hospital) for their valuable suggestions. We also thank Ms. Yuko Sugiura (Division of Dental Hygienists, Okayama University Hospital), Mr. Tomoaki Kuwano, Mr. Masaki Watanabe, and Ms. Naomi Kondo (Center for Innovative Clinical Medicine, Okayama University Hospital) for their excellent technical support. We are grateful to Ms. Eriko Yokoyama and Ms. Fumiko Tanabe (Department of Pathophysiology-Periodontal Science, Graduate School of Medicine, Dentistry, and Pharmaceutical Sciences, Okayama University) for their secretarial support. In addition, we wish to thank Mr. Tatsuya Ori, Mr. Narimichi Honda, Mr. Yuya Yamamoto, Ms. Akari Shimozono-Yamamoto, Mr. Yoshihiro Miura, Ms. Hiromi Managi, Ms. Emi Ohta (Sun Medical Co., Ltd.), Mr. Tomohisa Takagi, and Mr. Yuji Futamura (J Morita Corp.) for their support in the development of the Soft Protector CPC.

We would like to thank Editage (www.editage.com) for the English language editing.

References

1. Xia J, Tao X, Hu Q, Luo W, Tong X, Zhou G, et al. Expert consensus on the prevention and treatment of radiochemotherapy-induced oral mucositis. Int J Oral Sci. 2025;17: 54. doi: 10.1038/s41368-025-00382-8.

2. Bowen J, Cross C. The role of the innate immune response in oral mucositis pathogenesis. Int J Mol Sci. 2023;24: 16314. doi: 10.3390/ijms242216314.

3. Jasiewicz F, Qurban Z, Hughes C. Treatment-induced mucositis in oncology. Br J Hosp Med (Lond). 2022;83: 1-8. doi: 10.12968/hmed.2022.0324.

4. Nurhidayah I, Nurhaeni N, Allenidekania A, Gayatri D, Mediani HS. The effect of oral care intervention in mucositis management among pediatric cancer patients: An updated systematic review. J Multidiscip Healthc. 2024;17: 3497-3515. doi: 10.2147/JMDH.S467455.

5. Chan J, Filippi F, Filippi C. Clinical guidance for maintaining oral hygiene in patients undergoing chemotherapy or radiation therapy: A scoping review. Swiss Dent J. 2023;133: 368-379. doi: 10.61872/sdj-2023-06-01.

6. Colella G, Boschetti CE, Vitagliano R, Colella C, Jiao L, King-Smith N, et al. Interventions for the prevention of oral mucositis in patients receiving cancer treatment: Evidence from randomised controlled Trials. Curr Oncol. 2023;30: 967-980. doi: 10.3390/curroncol30010074.
7. European Oral Care in Cancer Group (EOCC). Oral care guidance. 1st Japanese ed.. Japanese Association of Supportive Care in Cancer Mucositis Subcommittee.
8. Multinational Association of Supportive Care in Cancer (MASCC)/International Society of Oral Oncology (ISOO). Evidence-based clinical practice guidelines for mucosal injury associated with cancer treatment. Japanese ed. MASCC/ISOO Mucositis Study Group; 2014.
9. Barasch A, Peterson DE. Risk factors for ulcerative oral mucositis in cancer patients: unanswered questions. Oral Oncol. 2003;39: 91-100. doi: 10.1016/s1368-8375(02)00033-7.
10. Boers-Doets CB, Epstein JB, Raber-Durlacher JE, Ouwerkerk J, Logan RM, Brakenhoff JA, et al. Oral adverse events associated with tyrosine kinase and mammalian target of rapamycin inhibitors in renal cell carcinoma: A structured literature review. Oncologist. 2012;17: 135-144. doi: 10.1634/theoncologist.2011-0111.

11. Heist RS, Sands J, Bardia A, Shimizu T, Lisberg A, Krop I, et al. Clinical management, monitoring, and prophylaxis of adverse events of special interest associated with datopotamab deruxtecan. Cancer Treat Rev. 2024;125: 102720. doi: 10.1016/j.ctrv.2024.102720.

Click here to access/download
Supporting Information
20251028_S1_Protocol_Japanese.docx

Click here to access/download
Supporting Information
20251028_S2_Protocol_English translation.docx

Supplementary File

Click here to access/download
Supporting Information
20251124_SPIRIT 2025 editable checklist_Omori.docx
