# Supplementary material for: A multicenter, randomized, parallel-group confirmatory study protocol to evaluate the efficacy of Soft Protector CPC, a novel oral mucosal protectant, in preventing oral mucositis and alleviating pain in patients with breast cancer
Source: PLoS One. 2026 Jun 4;21(6):e0350803. doi: 10.1371/journal.pone.0350803 (PMC13235912; doi:10.1371/journal.pone.0350803)
Supplement: Attachment — Submitted filename: 20260420_Reviewer comments & response_Omori_PLOSOne.docx [file pone.0350803.s007.docx]

***Journal Requirements:***

***When submitting your revision, we need you to address these additional requirements.***

***1. Please ensure that your manuscript meets PLOS ONE's style requirements, including those for file naming. The PLOS ONE style templates can be found at https://journals.plos.org/plosone/s/file?id=wjVg/PLOSOne_formatting_sample_main_body.pdf and https://journals.plos.org/plosone/s/file?id=ba62/PLOSOne_formatting_sample_title_authors_affiliations.pdf***

We thank the editor for this important comment. In accordance with the PLOS ONE style requirements, we have revised the manuscript formatting, including file naming, to ensure compliance with the journal’s guidelines and templates.

***2. Please provide a complete Data Availability Statement in the submission form, ensuring you include all necessary access information or a reason for why you are unable to make your data freely accessible. If your research concerns only data provided within your submission, please write "All data are in the manuscript and/or supporting information files" as your Data Availability Statement.***

We thank the editor for this important comment. In accordance with the journal’s instruction, we have provided the following complete Data Availability Statement in the submission form: “No datasets were generated or analyzed during the current study. All relevant data from this study will be made available upon study completion.

***3. We note that the grant information you provided in the ‘Funding Information’ and ‘Financial Disclosure’ sections do not match. When you resubmit, please ensure that you provide the correct grant numbers for the awards you received for your study in the ‘Funding Information’ section.***

We thank the editor for this important comment. In accordance with the editor’s suggestion, we have checked the funding information carefully and corrected the grant number(s) in the “Funding Information” section to ensure consistency with the “Financial Disclosure” section.

***4. Thank you for stating the following financial disclosure: This research is supported by the Japan Agency for Medical Development (AMED) under grant number JP25ck0106033. Please state what role the funders took in the study. If the funders had no role, please state: "The funders had no role in study design, data collection and analysis, decision to publish, or preparation of the manuscript." If this statement is not correct you must amend it as needed. Please include this amended Role of Funder statement in your cover letter; we will change the online submission form on your behalf.***

We thank the editor for this important comment. In accordance with the editor’s instruction, we have added the amended Role of Funder statement to the cover letter.

***5. Thank you for stating the following in the Competing Interests/Financial Disclosure section: The authors received financial support from the Japan Agency for Medical Research and Development (grant number JP25ck0106033). This study supported by San Medical Co., Ltd., for providing the investigational material (Soft Protector CPC). We note that you received funding from a commercial source: San Medical Co., Ltd., Please provide an amended Competing Interests Statement that explicitly states this commercial funder, along with any other relevant declarations relating to employment, consultancy, patents, products in development, marketed products, etc. Within this Competing Interests Statement, please confirm that this does not alter your adherence to all PLOS ONE policies on sharing data and materials by including the following statement: "This does not alter our adherence to PLOS ONE policies on sharing data and materials.” (as detailed online in our guide for authors*** [***http://journals.plos.org/plosone/s/competing-interests***](http://journals.plos.org/plosone/s/competing-interests)***). If there are restrictions on sharing of data and/or materials, please state these. Please note that we cannot proceed with consideration of your article until this information has been declared. Please include your amended Competing Interests Statement within your cover letter. We will change the online submission form on your behalf.***

We thank the editor for this important comment. In accordance with the editor’s instruction, we have amended the Competing Interests Statement to explicitly disclose the commercial support from San Medical Co., Ltd. and included the revised statement in the cover letter. We have also confirmed that this does not alter our adherence to all PLOS ONE policies on sharing data and materials.

***6. Please ensure that you refer to Figure 1 in your text as, if accepted, production will need this reference to link the reader to the figure.***

We thank the editor for this comment. In accordance with the editor’s suggestion, we have added a reference to Figure 1 in the main text.

***7. We note that Figure 2 in your submission contain copyrighted images. All PLOS content is published under the Creative Commons Attribution License (CC BY 4.0), which means that the manuscript, images, and Supporting Information files will be freely available online, and any third party is permitted to access, download, copy, distribute, and use these materials in any way, even commercially, with proper attribution. For more information, see our copyright guidelines:*** [***http://journals.plos.org/plosone/s/licenses-and-copyright***](http://journals.plos.org/plosone/s/licenses-and-copyright)***. We require you to either (1) present written permission from the copyright holder to publish these figures specifically under the CC BY 4.0 license, or (2) remove the figures from your submission:
a. You may seek permission from the original copyright holder of Figure 2 to publish the content specifically under the CC BY 4.0 license.
We recommend that you contact the original copyright holder with the Content Permission Form (***[***http://journals.plos.org/plosone/s/file?id=7c09/content-permission-form.pdf***](http://journals.plos.org/plosone/s/file?id=7c09/content-permission-form.pdf)***) and the following text:
“I request permission for the open-access journal PLOS ONE to publish XXX under the Creative Commons Attribution License (CCAL) CC BY 4.0 (***[***http://creativecommons.org/licenses/by/4.0/***](http://creativecommons.org/licenses/by/4.0/)***). Please be aware that this license allows unrestricted use and distribution, even commercially, by third parties. Please reply and provide explicit written permission to publish XXX under a CC BY license and complete the attached form.”
Please upload the completed Content Permission Form or other proof of granted permissions as an "Other" file with your submission.
In the figure caption of the copyrighted figure, please include the following text: “Reprinted from [ref] under a CC BY license, with permission from [name of publisher], original copyright [original copyright year].”
b. If you are unable to obtain permission from the original copyright holder to publish these figures under the CC BY 4.0 license or if the copyright holder’s requirements are incompatible with the CC BY 4.0 license, please either i) remove the figure or ii) supply a replacement figure that complies with the CC BY 4.0 license. Please check copyright information on all replacement figures and update the figure caption with source information. If applicable, please specify in the figure caption text when a figure is similar but not identical to the original image and is therefore for illustrative purposes only.***

We thank the editor for pointing this out. Following the editor’s instruction, we removed the copyrighted exterior photograph(s) from Figure 2 and revised the submitted figure to ensure compliance with PLOS ONE’s copyright and licensing requirements.

***8. We note that there is identifying data in the Supporting Information file <20251028_S1_Protocol_Japanese.docx, 20251028_S2_Protocol_English translation.docx>. Due to the inclusion of these potentially identifying data, we have removed this file from your file inventory. Prior to sharing human research participant data, authors should consult with an ethics committee to ensure data are shared in accordance with participant consent and all applicable local laws.
Data sharing should never compromise participant privacy. It is therefore not appropriate to publicly share personally identifiable data on human research participants. The following are examples of data that should not be shared:
-Name, initials, physical address
-Ages more specific than whole numbers
-Internet protocol (IP) address
-Specific dates (birth dates, death dates, examination dates, etc.)
-Contact information such as phone number or email address
-Location data
-ID numbers that seem specific (long numbers, include initials, titled “Hospital ID”) rather than random (small numbers in numerical order)
Data that are not directly identifying may also be inappropriate to share, as in combination they can become identifying. For example, data collected from a small group of participants, vulnerable populations, or private groups should not be shared if they involve indirect identifiers (such as sex, ethnicity, location, etc.) that may risk the identification of study participants.
Additional guidance on preparing raw data for publication can be found in our Data Policy (***[***https://journals.plos.org/plosone/s/data-availability#loc-human-research-participant-data-and-other-sensitive-data***](https://journals.plos.org/plosone/s/data-availability#loc-human-research-participant-data-and-other-sensitive-data)***) and in the following article:*** [***http://www.bmj.com/content/340/bmj.c181.long***](http://www.bmj.com/content/340/bmj.c181.long)***.
Please remove or anonymize all personal information (<specific identifying information in file to be removed>), ensure that the data shared are in accordance with participant consent, and re-upload a fully anonymized data set. Please note that spreadsheet columns with personal information must be removed and not hidden as all hidden columns will appear in the published file.***

We thank the editor for pointing this out. Following the editor’s instruction, we carefully reviewed the Supporting Information files, removed all potentially identifiable personal information, and prepared fully anonymized versions in accordance with participant privacy protection requirements.

***9.*** [***Please include captions for your Supporting Information files at the end of your manuscript, and update any in-text citations to match accordingly. Please see our Supporting Information guidelines for more information: http://journals.plos.org/plosone/s/supporting-information.***](http://track.editorialmanager.com/CL0/http:%2F%2Fjournals.plos.org%2Fplosone%2Fs%2Fsupporting-information./1/010f019c92f68acf-786b1867-fe73-4621-8773-46d290d433c7-000000/bdDjk0iQeMLoMvJZYeYzRD58QwxFaW2of2_SWQsL3n4=251)

We thank the editor for pointing this out. Following the editor’s instruction, we added the captions for the Supporting Information files at the end of the manuscript and revised the corresponding in-text citations to ensure consistency.

***10. If the reviewer comments include a recommendation to cite specific previously published works, please review and evaluate these publications to determine whether they are relevant and should be cited. There is no requirement to cite these works unless the editor has indicated otherwise.***

We thank the editor for this helpful comment. In accordance with the editor’s suggestion, we evaluated the publications recommended by the reviewers and incorporated the relevant references into the revised manuscript where appropriate.

***Reviewer #1: As the statistical reviewer I will focus on methods and reporting

Major
1) The power calculation is necessarily arbitrary. report as cohen's d for clarity, the effect size. is ICC expected since this is a multi centre trial? is there no chance of drop outs? that is not accounted in the power calculations.***

Thank you for your careful review and the insightful comments. We provide our responses to each point below.

i) The power calculation is necessarily arbitrary.

Response:

As described in the “Sample size and statistical analyses” section of the Materials and Methods, in our previous study, 58.3% (7/12) of patients in the group using Soft Protector CPC alongside oral care achieved the primary endpoint, compared to 0.0% (0/6) in the oral care-only group. Given the limited sample size of the previous exploratory study, the proportions for the power calculation of this trial were set at 50.0% for the intervention group and 25.0% for the control group. These values were established as clinically meaningful and more conservative expected group differences compared to the findings of the previous exploratory study.

Therefore, the sample size for this study was determined conservatively based on the results of the exploratory research and is not based on arbitrary assumptions. We have added the following description to the manuscript to clarify this point:

*Section: Sample size and statistical analyses*

*These values were set as clinically meaningful and more conservative expected group differences compared to the findings of the previous exploratory study.*

ii) Report as Cohen's d for clarity, the effect size.

Response:

Since the primary endpoint of this study is a binary variable, we calculated the effect size using Cohen’s h. Based on the aforementioned hypothesis, the effect size is h = 0.52, which corresponds to a medium effect size and is considered a clinically meaningful difference. We have added this information to the manuscript as follows:

*Section: Sample size and statistical analyses*

*Based on the aforementioned hypothesis, the effect size is Cohen’ s h = 0.52, corresponding to a medium effect size and suggesting a clinically meaningful difference.*

iii) Is ICC expected since this is a multi-centre trial?

Response:

The number of institutions participating in this study is small, and it is anticipated that there will be differences in the number of feasible participants between institutions. Therefore, we considered it difficult to appropriately adjust for the Intraclass Correlation Coefficient (ICC) during the sample size design phase. Furthermore, since the types of chemotherapy regimens for patients within an institution are not limited to a small number, the similarity between such patients is unlikely to be high. Consequently, the ICC is expected to be close to 0, and we believe that even if an adjustment were made, its impact would be minimal. We plan to evaluate the influence of inter-institutional differences during the analysis stage (see response to Reviewer#1 Major3)4)5)7) and Section Sample size and statistical analyses).

iv) Is there no chance of dropouts? That is not accounted in the power calculations.

Response:

In the previously exploratory study, no dropouts occurred, regardless of cancer type. It should be noted that during the middle of the evaluation period, some male cancer patients expressed difficulty with frequent visits due to their business work commitments. In contrast, female breast cancer patients were cooperative with the study and did not raise such concerns. Based on these experiences, we have designed this trial to limit the timing of product application and evaluation strictly to start date of the chemotherapy administration cycle, thereby eliminating unimportant hospital visits. We believe this makes dropouts for such reasons less likely to occur.

Furthermore, since the comparative evaluation period covers only one cycle of cancer treatment, mid-term dropout is unlikely. Additionally, not a single adverse event with a causal relationship to this product was observed in the exploratory study. For these reasons, we consider the dropout rate to be extremely low and do not account for its impact in the sample size design or statistical analysis.

However, in response to your comment, we have performed the following additional consideration as a precaution. The required sample size for this study is set at 154 cases with a power of 90%. Under a generally accepted power of 80%, the required sample size would be 116 cases. Therefore, although we consider such a situation unlikely, sufficient power would be maintained even if a dropout rate of approximately 24.6% (38 cases) were to occur.

***2) there is no mention of the consort statement and its relevance here.***

Response：

Thank you for this valuable comment. We have clarified the reporting framework by specifying the relevant reporting guidelines in the revised manuscript. Specifically, we have added a statement indicating that this study protocol was developed in accordance with the SPIRIT2025 statement, which is the appropriate reporting guideline for randomized clinical trial protocols. We have also noted that the trial results will be reported in accordance with the CONSORT guidelines. We have added this information to the manuscript as follows:

*Section: Materials and methods*

*This protocol was prepared in accordance with the SPIRIT 2025 guidelines. The completed SPIRIT checklist is provided as Supporting Information (S2 Checklist). The reporting of the trial results will follow the CONSORT guidelines.*

***3) i'd advise analyses to follow a regression framework so effect sizes and uncertainty can be easily quantified and reported - moving away a bit from p-values, they shouldn't be the focus.***

***4) what characteristics will be reported for each arm, on which balanced will be evaluated? what will the authors do if the randomisation has not ensured balanced across all covariates considering the small sample? and will missing data be dealt with (perhaps multiple imputation as a sensitiivty analysis)***

***5) again going back to expected heterogeniety, or not, across centres, the authors need to consider how this affects the study. in the power calculation but also in the analyses. in a regression framework one would expect a mixed effects model with a random intercept for centre.***

***7) any interventions (medications) need to be monitored and pehaps used in the regression model if there is no complete balance.***

Response: As points 3)4)5)7) are closely interrelated, we have addressed them collectively as follows:

We entirely agree that quantifying the effect of the study device through a regression model, which provides more information than simple hypothesis testing, is of great significance. By appropriately incorporating covariates that may influence the primary endpoint into the model, we can evaluate their specific impacts. We have planned a regression analysis that includes these covariates simultaneously, which also serves to adjust for potential heterogeneity in the distribution of these variables between the groups. At the planning stage, we have designated center, baseline Grade on Day 1 of the comparative period, and chemotherapy regimen as the covariates to be included in the model.

Since the number of participating centers is small (n=4), we believe it is more appropriate to treat center as a fixed effect. Similarly, baseline Grade will be treated as a fixed effect. For the overall analysis, this will be dichotomized as "Grade 0" or "Grade 1 or higher." However, for the "palliative effect" subset analysis, it will be incorporated into the model using the original Grades (1, 2, 3, and 4). Due to the wide variety of chemotherapy regimens, they will be planned to be treated as a random effect. The necessity of considering other covariates will be confirmed through a data review after the completed study. Potential candidates for such additional covariates include the presence or absence of candidiasis on Day 1 and the concomitant use of G-CSF preparations. These analyses are pre-specified in the Statistical Analysis Plan (SAP). However, considering the potential for multiplicity arising from the selection of an appropriate model through various covariate combinations, we have positioned these as secondary analyses.

Regarding missing data, we anticipate that the lack of the primary endpoint will not be frequent enough to significantly impact the overall study results. Therefore, we do not include procedures such as multiple imputation in the SAP. Our pre-specified approach for data handling is as follows: "Unless otherwise specified, no imputation for missing data will be performed, and the analysis will be conducted based on observed data. When calculating proportions, the denominator will be the Full Analysis Set (FAS). Consequently, unassessable cases will be included in the denominator, and an 'Unknown' category will be created and classified if necessary." However, if the data review confirms a clear need for imputation, we will revise the SAP and conduct additional sensitivity analyses using methods such as multiple imputation.

Furthermore, the primary analysis for the primary endpoint is planned as follows:

For the intervention and control groups during the comparative period, the following analyses are conducted:

(1) Primary Analysis

a) Cross-tabulations of each Grade at Day 1 of the comparative period versus the end of the comparative period (or at withdrawal) will be performed for each group.

b) For prevention of pain onset or pain improvement from Day 1 to the end of the comparative period (or at withdrawal), the frequency and proportion (%) will be calculated for each group, and the two-sided 95% confidence intervals (CIs) for the proportions will be estimated.

c) The point estimate and two-sided 95% CI for the difference in proportions between groups (intervention group minus control group) will be calculated. Additionally, Pearson’s chi-square test with Yates’ continuity correction will be applied.

The primary analysis described above does not account for factors that could be separately explained as covariate effects in the regression model analysis; instead, all variance is encompassed as intra-individual variation. Therefore, we consider this to be a conservative approach for evaluating the superiority of the device.

Given the above, since the plans to implement the model-based analysis approach as a secondary analysis and the procedures for handling missing data were not previously stated in the manuscript, we have added the following descriptions in light of your suggestions. Furthermore, we have supplemented the description of the primary analysis for the primary endpoint where the previous explanation was insufficient.

Regarding the model-based analysis for the primary endpoint, the following description has been added:

*Section: Sample size and statistical analyses*

*As a secondary analysis of the primary endpoint, we will estimate the risk difference in the proportion of patients between groups using a least-squares regression approach with robust standard errors [9]. The model includes center, baseline pain grade on Day 1 of the comparative period, and treatment group as fixed effects, with chemotherapy regimen treated as a random effect. From this model, we will report the point estimate for the treatment effect together with the 95% confidence interval and p-value.*

Regarding the handling of missing data, the following description has been added:

*Section: Sample size and statistical analyses*

*All analyses will be performed on the full analysis set without imputation of missing values. For proportions, the denominator will be the total number of participants in the analysis set, including those with missing or unevaluable data.*

Regarding the primary analysis of the primary endpoint, additional details have been provided where the previous description was insufficient (the added parts are underlined):

*Section: Sample size and statistical analyses*

*The primary endpoint will be the proportion of patients achieving prevention of the onset or alleviation of pain based on the subcriteria from the CTCAE v3.0 during the comparative period. The CTCAE grades at Day 1 and at the end of the comparative period will be descriptively summarized by treatment group, and a 95% confidence interval will be calculated by treatment group. The between-group difference in proportions and its two-sided 95% confidence interval will also be estimated. The superiority of Soft Protector CPC over the control treatment will be assessed using the Pearson’s chi-square test with Yates’ continuity correction at a two-tailed statistical significance level of 5%.*

***6) there are numerous secondary outcomes. analyses for these are likely to be underpowered and authors need to be clear these analyses will be described as exploratory.***

Response：

Thank you for this important comment. We agree that the study is not powered to detect statistically significant differences in the secondary outcomes. We have revised the manuscript to clarify this point and added the following statement:

*Section: Secondary endpoints*

*Secondary endpoints are pre-specified to support the interpretation of the primary endpoints; however, analyses of these endpoints will be considered exploratory, as the study is not powered for these outcomes.*

***Minor***

***1) is there a plan for safety to be monitored statistically? this relates to the fact there are no interim analyses planed.***

Response：

We thank the reviewer for this comment. The Soft Protector CPC is a non-invasive medical device designed for physical protection and, unlike systemic pharmacological interventions, carries a very low risk of serious adverse events. This is further supported by our previous exploratory study, in which only one adverse event was observed; notably, this event occurred prior to device application and was determined to have no causal relationship to the intervention, effectively meaning that no adverse events occurred in practice. Based on these considerations, we judged that formal statistical stopping criteria for safety were not warranted, and no interim analysis was planned. As the description in the manuscript was insufficient, we have revised the relevant section as follows:

*Section: Safety monitoring*

*Given the low-risk nature of the intervention and the absence of serious adverse events in our previous exploratory study, no formal interim analysis is planned.*

***Reviewer #2: GENERAL ASSESSMENT
This manuscript describes a multicenter, randomized, open-label controlled trial protocol evaluating the efficacy of the Soft Protector CPC for the prevention or alleviation of chemotherapy-induced oral mucositis in patients with breast cancer. The topic is clinically relevant, and the randomized design and inclusion of both clinician-assessed and patient-reported outcomes are strengths. However, the protocol raises substantial methodological concerns related to the definition of the primary endpoint, its alignment with the two study periods, and the justification of the sample size and statistical analysis, all of which affect the interpretability and confirmatory strength of the study.

MAJOR COMMENTS:

1. Primary endpoint definition and clinical relevance
The primary endpoint is defined as “the proportion of patients achieving prevention of the onset or alleviation of pain during the comparative period” and is planned to be analyzed as a binary outcome using a Pearson chi-square test. This definition raises important concerns. It combines two conceptually distinct phenomena: prevention of oral mucositis onset, which applies to the entire study population, and alleviation of pain, which is relevant only to the subset of patients who develop symptoms. Collapsing these constructs into a single binary outcome obscures clinical interpretation and makes patient classification unclear.
In addition, the protocol does not specify how “alleviation of pain” is operationally defined (e.g., magnitude of change, timing, or applicable patient subset), leaving this component open to subjective interpretation and inconsistent application. These issues are particularly relevant given that oral mucositis in breast cancer chemotherapy is of moderate incidence: published data suggest rates of approximately 20–40%, with severe cases (CTCAE grade ≥3) in fewer than 10% of patients (e.g., Elting et al. 2003 DOI: 10.1002/cncr.11671; Sonis, 2004 DOI: 10.1038/nrc1318; Lalla et al. 2014 DOI: 10.1002/cncr.28592). As many participants may never develop mucositis or pain, inclusion of pain alleviation within the primary endpoint risks inflating apparent success without clearly reflecting a preventive effect. The primary endpoint should therefore be redefined with clear operational criteria and aligned with the preventive nature of the study population.***

Response:

Thank you for your careful review and insightful comments. We respond to your points as follows:

i) Relationship between the study population and the primary endpoint:

As you pointed out, the distribution of oral mucositis severity associated with cancer chemotherapy is mostly Grade 0 (no symptoms) or Grade 1 in order of frequency, while Grade 2 is less common, and Grade 3 or higher rarely occurs. We anticipate a similar distribution in this study; therefore, we have not set any particular restrictions on the severity of symptoms prior to the start of the intervention in the target patients.

Next, the target of "symptomatic relief" is a subset of patients who have ongoing oral mucositis symptoms from previous chemotherapy, which is consistent with your understanding. However, the target of the "preventive effect" differs from your interpretation. This product can only be expected to have an indirect preventive sub-effect on the entire oral cavity through the sustained-release of component, CPC. By attaching the product to the tooth sites at risk, we expect a direct effect of the product in preventing the onset of stomatitis at those specific sites. Therefore, the "preventive effect" group is a patient subset consisting of those who are newly undergoing chemotherapy, or those who have not experienced oral mucositis symptoms at the relevant site in previous chemotherapy, or those whose symptoms have healed after experiencing them. There is no overlap between these two subsets, and together they constitute the entire study population.

We understand that it would be more desirable to conduct two separate studies with sufficient power for each of these two effects. Considering this alongside feasibility, the objective of the study as a whole is to evaluate the "overall effect" expected from this product, namely the prevention and symptomatic relief of oral mucositis. As a secondary analysis for the primary endpoint, subset analyses based on the pain grade on the first day of the comparative period (Grade 0 for the onset prevention subset and Grades 1, 2, and 3 for the pain alleviation subset) are planned to strictly distinguish between "preventive effect" and "alleviation of pain". Therefore, overestimation of the "preventive effect" due to the inclusion of the "alleviation of pain" effect cannot occur as a result of this sub-analysis.

As you noted, the severity and temporal changes of symptoms vary depending on the treatment regimen. In this study, to reduce bias based on regimens and patient characteristics as much as possible, we limited the subjects to breast cancer patients. However, even when limited to breast cancer, treatment regimens are diverse. On the other hand, limiting the study design to only a few types of regimens would reduce the feasibility of the study and limit the generalizability of the results. Therefore, the treatment regimens included in this study are limited to those that are frequently used at respective institutions. Furthermore, by performing randomization at each institution, we maintain a balance across different treatment regimens and ensure comparability between groups.

Furthermore, to minimize potential bias related to the timing of product application, the initiation of the test product is synchronized with the first day of each chemotherapy cycle.

Based on the above, since no specific conditions have been set regarding the degree of symptoms, there are no additional descriptions. On the other hand, regarding the target breast cancer chemotherapy regimens, the timing of product application, and the fact that "preventive effect" and "alleviation of pain" will be strictly distinguished and conducted as a secondary analysis of the primary endpoint, these explanations in the underlined parts above will be described in the manuscript.

*Section: Sample size and statistical analyses*

*Furthermore, subset analyses will be conducted to strictly distinguish between preventive and alleviating effects according to the baseline pain grade; specifically, Grade 0 will be categorized as the onset prevention subset, while Grades 1, 2, and 3 will be categorized as the pain alleviation subset.*

*Section: Randomization*

*To ensure comparability between the groups, the chemotherapies included in this study are limited to those frequently used at respective institutions. Furthermore, randomization stratified by institution is employed to maintain balance across different chemotherapies, thereby enhancing comparability between the groups.*

*Section: Assessment schedule (the underlined part indicates the corresponding revision.)*

*To minimize potential bias related to the timing of product application, the initiation of the test product will be synchronized with the first day of each chemotherapy cycle. In addition, the CTCAE and the 15-item oral health questionnaire of the European Organization For Research And Treatment Of Cancer [EORTC QLQ-OH15] used as endpoints are internationally recognized indicators that have also been adopted in clinical guidelines for the management of oral mucositis [7,8].*

ii) Definition of the primary endpoint

The definitions of each component, assessment methods (degree of change), and assessment timing in this study are as follows:

・Prevention of onset: Absence of oral mucositis (Grade 0) at the affected site both at the initiation of study procedure (i.e., Day1 of the comparative period) and at the end of the evaluation period (i.e., the end of the comparative period).

・Alleviating pain: in patients with oral mucositis of Grade 1, 2, or 3 at the initiation of the study procedure (i.e., Day1 of the comparative period), either a reduction of at least one grade or complete resolution of symptoms at the end of the evaluation period (i.e., the end of the comparative period).

・When the symptoms of grade 1 oral mucositis - according to the subcriteria from the Common Terminology Criteria for Adverse Events version 3 (CTCAE v3.0) - disappear, the patient’s condition is defined as “absence of onset (for convenience, Grade 0)” as a supplemental definition specific to this study.

・Assessment timing: At the completion timing of the comparative period.

・Assessment method: Physician assessment based on CTCAE v3.0.

Regarding the above, we have revised or added the underlined parts in the manuscript as follows:

*Section: Primary endpoint*

*Primary endpoint*

*The primary endpoint in this study will be oral mucositis as assessed by physicians according to the subcriteria from the CTCAE v3.0 during the comparative period. The primary endpoint is evaluated exclusively based on the data obtained during the randomized comparative period. While similar assessments are performed during the continuous period, it is treated as exploratory endpoint. We will also evaluate the prevention of pain onset or the alleviation of pain. The endpoints are defined as follows:*

*- Prevention of onset: Absence of oral mucositis (Grade 0) at the affected site both at the initiation of study procedure (i.e., Day1 of the comparative period) and at the end of the evaluation period (i.e., the end of the comparative period).*

*- Alleviating pain: in patients with oral mucositis of Grade 1, 2, or 3 at the initiation of the study procedure (i.e., Day1 of the comparative period), either a reduction of at least one grade or complete resolution of symptoms at the end of the evaluation period (i.e., the end of the comparative period).*

*- When the symptoms of grade 1 oral mucositis - according to the subcriteria from the Common Terminology Criteria for Adverse Events version 3 (CTCAE v3.0) - disappear, the patient’s condition is defined as “absence of onset (for convenience, Grade 0)” as a supplemental definition specific to this study.*

***2. Relationship between endpoints and study periods
The protocol distinguishes between a randomized “comparative period” and a subsequent “continuous period” during which all participants receive Soft Protector CPC. While this design may be ethically motivated, its analytical implications are not sufficiently clarified. The comparative period is the only phase that allows a randomized comparison and must therefore underpin all primary efficacy conclusions. Outcomes assessed during the continuous period, when no control group remains, cannot support causal inference and should be considered descriptive or exploratory.
However, the protocol does not clearly restrict the primary endpoint to the comparative period nor explicitly define the continuous period as exploratory, creating a risk of overinterpretation of non-randomized results. This ambiguity is further compounded by the unclear endpoint definitions, particularly if symptom alleviation is emphasized beyond the randomized comparison. The authors should clearly delineate the analytical roles of the two study periods and explicitly limit confirmatory conclusions to the comparative period.***
Response:

Thank you for this important comment. We agree that only the randomized comparative period allows for valid causal inference and should therefore underpin all confirmatory efficacy conclusions. We have revised the manuscript to clearly delineate the analytical roles of the two study periods and to avoid any potential overinterpretation of non-randomized results.

Specifically, we have clarified that the randomized comparative period constitutes the basis for confirmatory analyses, whereas the subsequent non-randomized continuous period is intended for exploratory purposes only. The following statements have been added:

*Section: Assessment schedule*

*“This randomized comparative period is the primary period for evaluating treatment efficacy and serves as the basis for confirmatory statistical analyses.”*

*“The data from this non-randomized continuous period will be used for exploratory purposes.”*

*Section: Primary endpoint*

*“The primary endpoint is evaluated exclusively based on the data obtained during the randomized comparative period. While similar assessment is performed during the continuous period, it is treated as exploratory endpoint.”*

*Section: Sample size and statistical analyses*

*“This confirmatory analysis is restricted to the comparative period, while the continuous period will be analyzed for exploratory purposes.”*

***3. Statistical analysis inconsistencies
The sample size calculation is based on the primary endpoint described above and on a Pearson chi-square test comparing proportions between groups. The validity of this approach depends on a clearly defined binary outcome with a well-specified underlying probability structure. Given the composite and ambiguously defined nature of the primary endpoint, it is unclear how the assumed response rates used for the power calculation were derived or justified. In particular, the protocol appears to assume a relatively high success rate in the intervention group (>50%), which is difficult to reconcile with the moderate incidence of chemotherapy-induced oral mucositis in breast cancer patients unless the endpoint definition is overly broad.
Moreover, the chi-square–based power calculation implicitly assumes a uniform binary outcome assessed over a comparable observation window for all participants. This assumption is questionable in light of the heterogeneous occurrence of mucositis, the mixture of preventive and symptom-based components within the endpoint, and the variability in chemotherapy cycle length across patients. Consequently, the sample size justification lacks a transparent clinical and statistical foundation.
The protocol further specifies Pearson’s chi-square test as the sole inferential method for evaluating the primary endpoint while simultaneously stating an intention to demonstrate superiority of the intervention. The use of superiority language implies a directional hypothesis, which is conceptually inconsistent with a non-directional testing framework unless explicitly justified. Additionally, given the anticipated event rates and the composite nature of the endpoint, some contingency table cells may have small expected counts, potentially violating chi-square test assumptions. The authors should therefore clarify the intended hypothesis structure, ensure consistency between hypothesis, statistical test, and power calculation, and provide a clear justification for the chosen analytical approach.***

Thank you for your valuable comments. We recognize that our previous descriptions of the statistical analyses were insufficient, and we have revised the manuscript accordingly to address the points you raised.

As several of your comments overlap with those provided by Reviewer #1, we have addressed these points in detail in our response to Reviewer #1 to avoid unnecessary repetition. We would be grateful if you could kindly refer to that response for further clarification.

In addition, we provide the following supplementary explanations regarding items not covered in our response to Reviewer #1:

We will respond to your comments in two parts.

i) Regarding the comment, “The sample size calculation is based on the primary endpoint described above and on a Pearson chi-square test comparing proportions between groups. The validity of this approach depends on a clearly defined binary outcome with a well-specified underlying probability structure. Given the composite and ambiguously defined nature of the primary endpoint, it is unclear how the assumed response rates used for the power calculation were derived or justified. In particular, the protocol appears to assume a relatively high success rate in the intervention group (>50%), which is difficult to reconcile with the moderate incidence of chemotherapy-induced oral mucositis in breast cancer patients unless the endpoint definition is overly broad.”.

Although this overlaps with our response to Reviewer #1 (Major Comment 1), the rationale for the sample size determination is as follows.

As described in the “Sample size and statistical analyses” section of the Materials and Methods, in our previous study, 58.3% (7/12) of patients in the group using Soft Protector CPC alongside oral care achieved the primary endpoint, compared to 0.0% (0/6) in the oral care-only group. Given the limited sample size of the previous exploratory study, the proportions for the power calculation of this trial were set at 50.0% for the intervention group and 25.0% for the control group. These values were established as clinically meaningful and more conservative expected group differences compared to the findings of the previous exploratory study.

In addition, the relationship between the study population and the primary endpoint, as well as the definition of the primary endpoint, are addressed in our response to Reviewer #2 (Major Comment 1), and we kindly ask you to refer to that section for further details.

ii) Regarding the comment, “Moreover, the chi-square–based power calculation implicitly assumes a uniform binary outcome assessed over a comparable observation window for all participants. This assumption is questionable in light of the heterogeneous occurrence of mucositis, the mixture of preventive and symptom-based components within the endpoint, and the variability in chemotherapy cycle length across patients. Consequently, the sample size justification lacks a transparent clinical and statistical foundation.

The protocol further specifies Pearson’s chi-square test as the sole inferential method for evaluating the primary endpoint while simultaneously stating an intention to demonstrate superiority of the intervention. The use of superiority language implies a directional hypothesis, which is conceptually inconsistent with a non-directional testing framework unless explicitly justified. Additionally, given the anticipated event rates and the composite nature of the endpoint, some contingency table cells may have small expected counts, potentially violating chi-square test assumptions. The authors should therefore clarify the intended hypothesis structure, ensure consistency between hypothesis, statistical test, and power calculation, and provide a clear justification for the chosen analytical approach.”.

Although this overlaps with our responses to Reviewer #1 (Major Comments 3, 4, 5, and 7), the planned analysis of the primary endpoint is as follows. Please refer to our responses to Reviewer #1 (Major Comments 3, 4, 5, and 7) for details regarding the supplementary analyses.

Analysis of the primary endpoint:

For the intervention and control groups during the comparative period, the following analyses are conducted:

Primary Analysis

a) Cross-tabulations of each Grade at Day 1 of the comparative period versus the end of the comparative period (or at withdrawal) will be performed for each group.

b) For prevention of pain onset or pain improvement from Day 1 to the end of the comparative period (or at withdrawal), the frequency and proportion (%) will be calculated for each group, and the two-sided 95% confidence intervals (CIs) for the proportions will be estimated.

c) The point estimate and two-sided 95% CI for the difference in proportions between groups (intervention group minus control group) will be calculated. Additionally, Pearson’s chi-square test with Yates’ continuity correction will be applied.

Secondary analysis

As a secondary analysis of the primary endpoint, we will estimate the risk difference in the proportion of patients between groups using a least-squares regression approach with robust standard errors (Cheung, 2007). The model includes center, baseline pain grade on Day 1 of the comparative period, and treatment group as fixed effects, with chemotherapy regimen treated as a random effect. From this model, we will report the point estimate for the treatment effect together with its 95% confidence interval and p-value. Furthermore, subset analyses will be conducted to strictly distinguish between preventive and palliative effects according to the baseline pain grade; specifically, Grade 0 will be categorized as the onset prevention subset, while Grades 1, 2, and 3 will be categorized as the pain alleviation subset.

Thus, in the primary analysis, we adopt a conservative approach that does not explicitly adjust for specific factors (covariates), while in the secondary analyses, we plan to use regression models to appropriately account for the heterogeneity in patient characteristics and variability due to chemotherapies, as raised by the reviewer. In response to your comments, we have revised the manuscript to address sections where the description was insufficient. We have revised or added the underlined parts in the manuscript as follows:

*Section: Sample size and statistical analyses*

*The primary endpoint will be the proportion of patients achieving prevention of the onset or alleviation of pain based on the subcriteria from the CTCAE v3.0 during the comparative period. The CTCAE grades at Day 1 and at the end of the comparative period will be descriptively summarized by treatment group, and a 95% confidence interval will be calculated by treatment group. The between-group difference in proportions and its two-sided 95% confidence interval will also be estimated. The superiority of Soft Protector CPC over the control treatment will be assessed using the Pearson’s chi-square test with Yates’ continuity correction at a two-tailed statistical significance level of 5%.*

*As a secondary analysis of the primary endpoint, we will estimate the risk difference in the proportion of patients between groups using a least-squares regression approach with robust standard errors (Cheung, 2007). The model includes center, baseline pain grade on Day 1 of the comparative period, and treatment group as fixed effects, with chemotherapy regimen treated as a random effect. From this model, we will report the point estimate for the treatment effect together with the 95% confidence interval, Cohen’s d and p-value. Furthermore, subset analyses will be conducted to strictly distinguish between preventive and alleviating effects according to the baseline pain grade; specifically, Grade 0 will be categorized as the onset prevention subset, while Grades 1, 2, and 3 will be categorized as the pain alleviation subset.*

Response to Reviewer Comments Regarding the consistency between the superiority hypothesis and the non-directional test: “The protocol further specifies Pearson’s chi-square test as the sole inferential method for evaluating the primary endpoint while simultaneously stating an intention to demonstrate superiority of the intervention. The use of superiority language implies a directional hypothesis, which is conceptually inconsistent with a non-directional testing framework unless explicitly justified.” In this study, while "superiority" is defined as the clinical hypothesis, we have adopted a two-sided test (alpha = 0.05) for the statistical analysis. This approach is explicitly described in the ICH E9 guidelines established by academia and regulatory authorities. We believe that our plan is appropriate as it complies with these internationally recognized guidelines.

Regarding the small expected counts and the violation of chi-square assumptions: “given the anticipated event rates and the composite nature of the endpoint, some contingency table cells may have small expected counts, potentially violating chi-square test assumptions. The authors should therefore clarify the intended hypothesis structure, ensure consistency between hypothesis, statistical test, and power calculation, and provide a clear justification for the chosen analytical approach.” In this study, although the marginal frequencies of the contingency table are not small, we have adopted Pearson’s chi-square test with Yates’ continuity correction to account for cases where some cells may have small expected counts.

Yates’ continuity correction is a widely used method to correct the overestimation of the test statistic when expected frequencies are small in a 2×2 contingency table, thereby appropriately controlling the Type I error rate. We believe that this is a statistically appropriate approach in the context of this study.

***MINOR COMMENTS:
1. Different CTCAE versions (v3.0 and v5.0) are referenced; a single version should be specified and consistently applied.***

**Response:**

Thank you for this important comment. We agree that the use of different CTCAE versions should be explained more clearly to avoid confusion. In this protocol, CTCAE v3.0 is used for the **primary endpoint,** because it is consistent with the assessment framework used in our previous exploratory study and with the pre-specified confirmatory endpoint of the present trial. CTCAE v5.0 is used only for **secondary endpoint assessments** of oral mucositis and oral pain as a complementary and contemporary grading framework.

To make this distinction explicit and consistent throughout the manuscript, we have revised the relevant text in the Abstract, Introduction, and Endpoints sections to clearly state that **CTCAE v3.0 is used for the primary endpoint,** whereas **CTCAE v5.0 is used only for selected secondary endpoints.**

*Section: Introduction
The incidence and severity of oral mucositis will be assessed primarily using the Common Terminology Criteria for Adverse Events (CTCAE) v3.0 for the confirmatory primary endpoint, while CTCAE v5.0 will be used for selected secondary assessments of oral mucositis and oral pain.*

*Section: Primary endpoint
The primary endpoint in this study will be oral mucositis as assessed by physicians according to the subcriteria from the CTCAE v3.0 during the comparative period.*

***2. The procedures for screening, enrollment, and allocation concealment are insufficiently described and should be clarified.***

**Response:**

Thank you for this helpful comment. We agree that the sequence of screening, enrollment, and randomization should be described more explicitly. We have therefore revised the manuscript to clarify that written informed consent is obtained first, followed by screening and confirmation of eligibility according to the inclusion and exclusion criteria. Participants are then formally registered only after completion of the pre-registration examinations and final confirmation of eligibility, after which they are randomized to the intervention or control group.

We have also clarified the description of allocation concealment. Because this is an open-label trial, disclosure of the detailed randomization algorithm in the protocol could increase the risk of predicting group assignment. Therefore, the detailed allocation algorithm is documented in a separate specification and will remain confidential until completion of participant allocation.

*Section: Assessment schedule
Written informed consent will be obtained from all participants by principal investigator and co-investigators, and eligibility will be confirmed according to the inclusion and exclusion criteria. The participants will be registered after pre-registration examinations (screening tests) and final confirmation of eligibility. Enrolled participants will then be randomly assigned to either the intervention group (oral care with the application of the Soft Protector CPC, Fig 2) or the control group (oral care alone during the comparative period).*

*Section: Randomization
Prior to study initiation, participants will be randomly assigned in a 1:1 ratio to the intervention and control groups. Randomization will be conducted using a stratified block method with study institution as the stratification factor.*

*To ensure comparability between the groups, the chemotherapies included in this study are limited to those frequently used at respective institutions. Furthermore, randomization stratified by institution is employed to maintain balance across different chemotherapies, thereby enhancing comparability between the groups.*

*Because this is an open-label trial, the detailed allocation algorithm will be documented in a separate specification that will remain confidential until completion of participant allocation.*

***3. The standard of oral care provided to the control group should be more precisely defined to ensure consistency across centers.***

**Response:**

Thank you for this important comment. We agree that the standard oral care provided to the control group should be more precisely described to improve reproducibility and consistency across centers. We have therefore revised the manuscript to clarify that oral care is treated as a standardized background intervention across participating institutions. We also specified that oral care primarily consists of oral moisturization and routine supportive oral care according to the study procedure, and that the use of rescue treatments and other concomitant supportive measures will be recorded throughout the study.

*Section: Control group / Standardized oral care
During the comparative period, participants assigned to the control group will receive standardized oral care alone and will not receive Soft Protector CPC. After completion of the comparative period, they will enter the continuous period, during which Soft Protector CPC will be added to the same oral care regimen. Device application, removal, and discontinuation procedures during the continuous period will be identical to those used in the intervention group.*

*Oral care is regarded as a background intervention in both groups and will be provided according to a standardized study procedure across participating centers. Oral care will primarily consist of oral moisturization, and routine supportive oral care judged appropriate for the study setting. Any rescue treatment or additional oral supportive care, including oral steroid ointment, Episil^®^ oral liquid, and analgesic use, will be recorded throughout the study. Adherence to the assigned intervention will be monitored using physician records and participant-reported diaries.*

***4. The duration of participation per patient and the definition of study completion should be explicitly stated.***

**Response:**

Thank you for this useful comment. We agree that the duration of participation for each patient and the definition of study completion should be stated more explicitly. We have therefore revised the manuscript to clarify that each participant will generally be followed for two treatment periods: the randomized comparative period (1st course) and the continuous period (2nd course). Each period corresponds to one chemotherapy cycle and will generally last 14, 21, or 28 days, depending on the regimen.
We have also clarified that study completion for an individual participant is defined as completion of the end-of-treatment assessment at the end of the continuous period, or completion of the final assessment at the time of early discontinuation when applicable.

*Section: Assessment schedule
During the randomized comparative period (1st course), the intervention group will receive oral care plus Soft Protector CPC, whereas the control group will receive standardized oral care alone. This randomized comparative period is the primary period for evaluating treatment efficacy and serves as the basis for confirmatory statistical analyses.*

*After completion of all assessments in the comparative period, all participants, regardless of their original allocation, will proceed to the continuous period (2nd course), during which Soft Protector CPC will be added to oral care in both groups. The data from this non-randomized continuous period will be used for exploratory purposes.*

*Each treatment period will generally be set to 21 days; however, if chemotherapy is administered in cycles, the duration of one cycle (14, 21, or 28 days) will be used. To minimize potential bias related to the timing of product application, the initiation of the test product will be synchronized with the first day of each chemotherapy cycle.*
*Study completion for an individual participant is defined as completion of the planned assessment at the end of the continuous period or, in cases of early discontinuation, completion of the final assessment performed at the time of discontinuation.*

***CONCLUSION
This protocol addresses an important clinical question and has the potential to generate useful evidence. However, substantial clarification is required regarding the definition of the primary endpoint, its relationship to the study periods, and the justification of the statistical analysis and sample size. Addressing these issues will significantly strengthen the methodological rigor and interpretability of the trial.***

Thank you for your thoughtful overall assessment of our protocol and for recognizing its clinical relevance and potential value. We also appreciate your important methodological concerns regarding the definition of the primary endpoint, its relationship to the study periods, and the justification of the statistical analysis and sample size.

In response, we have revised the manuscript to clarify these key issues. Specifically, we have more explicitly defined the primary endpoint and its operational criteria, clarified that the confirmatory analysis is restricted to the randomized comparative period while the continuous period is exploratory, and strengthened the description and justification of the statistical analysis and sample size determination. We have also added further details regarding the primary analysis, model-based secondary analysis, and the handling of missing data. We believe that these revisions have substantially improved the methodological clarity, rigor, and interpretability of the protocol.

***Reviewer #3: All detailed comments are provided in the attached PDF document of the submitted manuscript.

1. The main concern relates to the description of all interventions planned in this study. Currently, the descriptions are vague and insufficiently detailed, and they should be more clearly and comprehensively reported. In particular, the protocol should explicitly describe how the tested device will be applied in the clinical setting, in accordance with the TIDieR checklist, to ensure transparency, reproducibility, and scientific rigor.***

**Response:**

Thank you for this important comment. We agree that the intervention procedures should be described in greater detail to improve transparency and reproducibility. In response, we have substantially revised the **Treatment** section in accordance with the principles of the TIDieR checklist. Specifically, we have clarified **what** the intervention is, **where** it is applied, **who** performs the procedure, **how** it is applied, and **how long** it is maintained. We have also clarified the procedure for removal, reapplication, and discontinuation.

In the revised manuscript, we now explicitly state that Soft Protector CPC is applied **to teeth that are likely to cause mucosal injury**, rather than directly to the oral mucosa, and we describe the clinical procedure in greater detail.

***2. Please explain more in detail what will be the difference in interventions in the continuous period between groups. It is also not clear from the Figure 3 what is the difference. What does it mean (Add-on)?***

Response:

Thank you for pointing this out. We agree that the difference between groups during the continuous period was insufficiently explained. We have revised the manuscript and Figure 3 legend to clarify that:

1. during the comparative period, the intervention group receives oral care plus Soft Protector CPC, whereas the control group receives oral care alone; and
2. during the continuous period, both groups receive oral care plus Soft Protector CPC.
   Thus, the term “Add-on” refers to the addition of Soft Protector CPC to the background oral care regimen. We have also clarified in the manuscript that the continuous period is non-randomized and exploratory and is not used for confirmatory causal inference.

***3. Will all patients receive the same type of chemotherapy? If not, will the type of chemotherapy administered be monitored? It is also important to assess its impact on the oral mucosa and on patients’ subjective symptoms (e.g. pain).***

**Response:**

Thank you for this valuable comment. Not all patients will receive the same chemotherapy regimen. In this study, eligible regimens are limited to those that are commonly used at each participating institution. We have clarified this point in the revised manuscript.

We also confirm that the chemotherapy regimen administered to each patient will be recorded and monitored. In addition, its potential impact on oral mucositis and pain will be considered in the planned analyses. Specifically, chemotherapy regimen is addressed in the subgroup analyses and is also incorporated into the model-based secondary analysis of the primary endpoint.

***4. It is not clear whether Soft Protector CPC is a device intended to be applied to the teeth or to the oral mucosa. Based on the current treatment description, it appears that the device is applied to the teeth. The clinical application of this device should be described in greater detail to ensure scientific and clinical reproducibility. It would be advisable to describe the entire clinical procedure in accordance with the TIDieR checklist (https://www.equator-network.org/wp-content/uploads/2014/03/TIDieR-Checklist-PDF.pdf) for all interventions applied in this study.***

**Response:**

Thank you for this important comment. We apologize that this point was not sufficiently clear in the previous version. Soft Protector CPC is **applied to the teeth,** not directly to the oral mucosa. Its purpose is to protect the oral mucosa from mechanical irritation caused by teeth that are likely to contact and injure the mucosa.

To improve reproducibility, we have revised the manuscript to describe the application procedure in greater detail, including identification of the target teeth, operator qualification, use of the bonding agent, direct placement of the material, removal of excess material, occlusal confirmation, light curing, and management during the treatment period. We have also revised the intervention description in line with the TIDieR framework.

***5. The same comment applies as for the treatment group. Oral care should also be considered an intervention (gold standard) and, therefore, should be clearly described and appropriately monitored.***

**Response:**

Thank you for this helpful comment. We agree that oral care constitutes an important background intervention and should be described more explicitly. In response, we have expanded the description of the oral care procedure in the revised manuscript and clarified that oral care is provided in a standardized manner across study periods and centers. We have also clarified how adherence to oral care and use of concomitant supportive measures, including rescue medications, are recorded and monitored during the study.

This revision was made to ensure that both the investigational intervention and the background oral care are sufficiently described for reproducibility and interpretation.
